# Genomic Characterization of Sixteen *Yersinia enterocolitica*-Infecting Podoviruses of Pig Origin

**DOI:** 10.3390/v10040174

**Published:** 2018-04-03

**Authors:** Mabruka Salem, Mikael Skurnik

**Affiliations:** 1 Department of Bacteriology and Immunology, Medicum, Research Programs Unit, Immunobiology, University of Helsinki, 00014 Helsinki, Finland; Mabruka.salem@helsinki.fi; 2 Department of Microbiology, Faculty of Medicine, University of Benghazi, Benghazi 16063, Libya; 3 Division of Clinical Microbiology, Helsinki University Hospital, HUSLAB, 00029 Helsinki, Finland

**Keywords:** phages, *Yersinia enterocolitica*, lipopolysaccharide, T7-like phages, Podoviruses, *Autographivirinae*, pig stool, terminal repeats

## Abstract

*Yersinia enterocolitica* causes enteric infections in humans and animals. Human infections are often caused by contaminated pork meat. *Y. enterocolitica* colonizes pig tonsils and pigs secrete both the human pathogen and its specific bacteriophages into the stools. In this work, sixteen *Y. enterocolitica*—infecting lytic bacteriophages isolated from pig stools originating from several pig farms were characterized. All phages belong to the *Podoviridae* family and their genomes range between 38,391–40,451 bp in size. The overall genome organization of all the phages resembled that of T7-like phages, having 3–6 host RNA polymerase (RNAP)-specific promoters at the beginning of the genomes and 11–13 phage RNAP-specific promoters as well as 3–5 rho-independent terminators, scattered throughout the genomes. Using a ligation-based approach, the physical termini of the genomes containing direct terminal repeats of 190–224 bp were established. No genes associated with lysogeny nor any toxin, virulence factor or antibiotic resistance genes were present in the genomes. Even though the phages had been isolated from different pig farms the nucleotide sequences of their genomes were 90–97% identical suggesting that the phages were undergoing microevolution within and between the farms. Lipopolysaccharide was found to be the surface receptor of all but one of the phages. The phages are classified as new species within the *T7virus* genus of *Autographivirinae* subfamily.

## 1. Introduction

*Yersinia enterocolitica* is a foodborne pathogen, causing yersiniosis; a zoonotic infection manifested by diarrhea, fever, and lymphadenitis. In some cases the infection is complicated by reactive arthritis [1]. Yersiniosis was estimated as the third most common zoonotic infectious disease in Europe in 2010 [2]. *Y. enterocolitica* serotype O:3 was the most common causative agent of yersiniosis in Finland [3]. *Y. enterocolitica* is generally susceptible to all antimicrobials except ampicillin [4,5]; however, resistance of some *Y. enterocolitica* strains to antibiotics has been reported [6,7].

Bacteriophages (phages) are bacterial viruses which are regarded as the most abundant entities in the universe with an estimation of 10^31^ virions in the biosphere [8]. As a consequence of increased antibiotic resistance, interest to use phages as antibacterial agents has also increased. Phage therapy is routinely being used to treat bacterial infections in Georgia, Russia and Poland [9,10]. No genes characteristic for lysogenic phages nor any toxin, virulence factor or antibiotic resistance genes should be carried by phages intended for therapeutic purposes; therefore, complete characterization and genome analysis of phages is a prerequisite [11].

The number of sequenced phage genomes increases on almost daily basis [8]; however, only a few phages infecting *Y. enterocolitica* have been studied. These include ϕYeO3-12 [12,13,14] and vB_YenP_AP5 [15], which are both lytic *Y. enterocolitica* O:3-specific T3 and T7-related podoviruses, respectively. Phage ϕR1-37 is a lytic myovirus which has a broader host range that includes in addition to many *Y. enterocolitica* serotypes also some *Y. intermedia* and *Y. similis* strains [16]. Another broad-host range phage for the genus *Yersinia*, is PY100 which is a lytic myovirus isolated from a pig manure in Germany [17]. Phages vB_YenM_TG1 and vB_YenM_ϕR1-RT are lytic myoviruses with relatively narrow host range that are able to infect *Y. enterocolitica* serotypes O:3, O:9 and O:5,27 strains [18].

We recently isolated *Yersinia*-specific phages from 90 out of 793 pig stool samples collected from different pig farms in Finland [19]. Based on host range analysis and the restriction enzyme digestion profiles, 19 different phages were chosen for further studies to represent the 90 isolates [19]. Electron microscopy revealed that 16 of the phages belong to the *Podoviridae*, and three, to the *Myoviridae* family. In this work, we have carried out a detailed characterization of the 16 *Podoviridae* phages.

## 2. Materials and Methods

### 2.1. Bacterial Strains, Phages, Media and Growth Conditions

The bacterial strains used in this work are described in Table 1. The phages were isolated from pig stool samples collected from different pig farms in Finland. These phages were enriched and purified according to the methods described earlier [19]. Lysogeny broth (LB), lysogeny agar (LA) plates (LB with 1.5% agar) and soft agar (LB with 0.4% agar) were the media used for propagation of bacteria and phages throughout this work. Chloramphenicol and kanamycin (20 µg/mL in liquid and 100 µg/mL in solid media) were added when required. The incubations of all the described experiments were at room temperature (RT) and overnight unless mentioned otherwise.

### 2.2. Electron Microscopy

To increase the phage concentration, phage particles were centrifuged at 16,000× *g* for 90 min at 4 °C using an Eppendorf centrifuge (5415R, rotor model 3328, Enfield, NJ, USA). The phage pellets were suspended in 0.1 M ammonium acetate. A drop of the phage suspension was deposited on a carbon-coated Formvar film on copper grid for one minute and then removed with a filter paper, followed by negative staining with 1% uranyl acetate (pH about 4.2) for 30 s, the excess dye was then removed by a filter paper. The phage particles were then examined under a transmission electron microscope (JEOL JEM-1400, Tokyo, Japan, 80 kV), using an Olympus Morada CCD-kamera, operating at iTEM software (EMSIS GmbH, Muenster, Germany).

### 2.3. Isolation of Phage DNA

Phage DNA was isolated following the protocol of the Invisorb^®^ Spin Virus DNA Mini Kit (Stratec, Berlin, Germany) or using the protocol described earlier [19].

### 2.4. Genome Sequencing, Assembly and Bioinformatics

The genomes of 16 pig stool phages (collectively called fPS-phages, Table 2) were sequenced by Illumina Miseq at the Institute for Molecular Medicine Finland (FIMM) [25]. The raw sequence data for each phage was then subjected to the A5 de novo assembly pipeline [26] and alignment using the computers at the Centers for Scientific Computing [27]. The genome sequences were aligned using the multiple sequence alignment program MAFFT [28] at the Chipster platform [29]. The annotations were performed using RAST server [30] and manually annotated using the Artemis genome browser and annotation tool [31]. Some RAST-identified open reading frames (ORFs) missed a good ribosomal binding site (RBS) and were corrected to a start codon with an appropriately located RBS. Nucleotide and amino acid sequence identities were determined using BLASTN and BLASTP [32], respectively. HHpred server [33] was also used for protein homology detection. Whole genome alignments were visualized using Geneious R10 software version 10.0.2. (Biomatters Ltd., Auckland, New Zealand). A search for transfer RNA (tRNA) genes was done using tRNAScan-SE [34]. The phage RNAP-specific promoters were identified using PHIRE program [35], and the putative transcriptional terminators were identified using ARNold software (http://rna.igmors.u-psud.fr/). The promoter consensus sequences were generated using the sequence logo generator (WebLogo) [36]. The host RNAP promoters were identified using BPROM [37]. All the identified promoter and terminator sequences were further verified manually using the Artemis. The phylogenetic trees for the amino acid sequences of the DNA ligase, RNAP, and capsid proteins were constructed using the service at Phylogeny.fr [38]. The phylogenetic tree of the whole genome sequences were generated by VICTOR [39].

### 2.5. Determination of the Genome Ends

**Figure 1:**
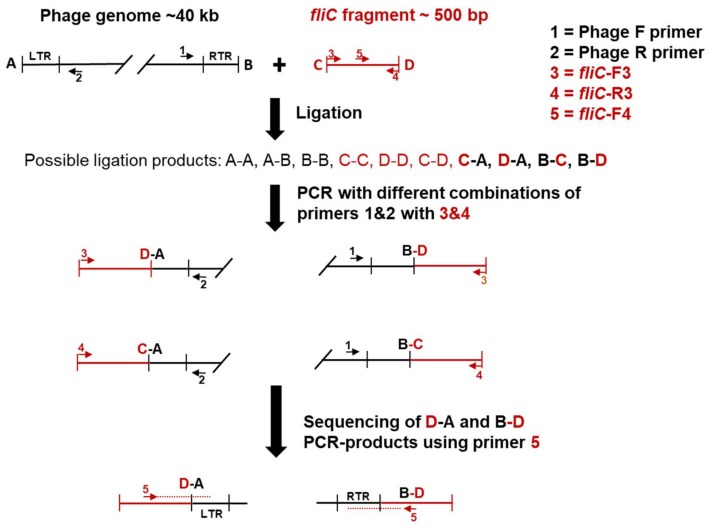
The ligation-polymerase chain reaction (PCR) strategy to determine the physical ends of bacteriophage genomes. The *fliC* fragment, its primers and directions are shown in red, and the phage genome, its primers and directions, in black. The predicted ends of the phage genomes and the *fliC* fragment are labeled by A & B, and C & D, respectively. First, the *fliC* gene fragment is blunt-end-ligated with phosphorylated intact phage genomic DNA. The ligation mixture is used as a template in PCR using *fliC* and phage specific primer pairs. The desired PCR-amplified ligation products are shown in bold. Finally, the D-A and B-D PCR-products are sequenced with a nested *fliC* specific primer *fliC*-F4.

The strategy to identify the physical ends of the genomes is outlined in Figure 1. First, the phage DNA was phosphorylated by adding 1 µL of T4 polynucleotide kinase (PNK) 10 U/µL (Thermo Scientific, Vilnius, Lithuania), 1 µL of 10 mM adenosine triphosphate (ATP) (New England BioLabs, Ipswich, MA, USA), 5 µL of 10× PNK buffer A (Thermo Scientific, Vilnius, Lithuania) and ~800 ng of phage DNA, in a total reaction volume of 50 µL. The mixture was incubated for 45 min at 37 °C. The kinase was then inactivated at 75 °C for 15 min. Ligation of the phosphorylated DNA to a 500 bp fragment of the *fliC* gene of *Y. enterocolitica* O:3 was performed in a 30 µL reaction mixture containing ~400 ng of the phosphorylated DNA, ~40 ng of *fliC* gene fragment, 2 µL of 50% (*w*/*v*) PEG 4000 (Thermo Scientific), 3 µL of 10 × T4 µL DNA Ligase buffer and 1 µL T4 DNA ligase (New England BioLabs). This mixture was incubated for 2 h at RT. The ligation mixture was then used as a template in PCR that was carried out in a total volume of 50 µL containing 5 µL of 10× buffer for DyNAzyme DNA polymerase (Thermo Scientific), 200 µM dNTPs (Bioline, London, UK), 1 µL of 1:10 diluted *fliC*-ligated DNA mixture and 2U of DyNAzyme II DNA polymerase (Thermo Scientific, Vilnius, Lithuania). For each PCR run, four different phage and *fliC*-specific primer combinations were used (Figure 1, Table 3). The PCR program contained a 3 min denaturation step at 95 °C, followed by 30 cycles of 30 s denaturation at 95 °C, 30 s annealing at 55–58 °C, and 60 s extension at 72 °C. This was followed by a 5 min extension at 72 °C.

The resulting PCR products were analyzed in 0.8% agarose gel (BMA, Rockland, ME, USA) running at 100 V for 50 min. Then, the correct-sized PCR products were gel-purified from eight parallel PCRs using a gel purification kit (E.Z.N.A Gel Purification kit, Omega Bio-Tek, Inc., Norcross, GA, USA) after running the combined samples in a 1% low melting agarose gel (BMA, Rockland, ME, USA). DNA concentration and quality were determined using the NanoDrop spectrophotometer ND-1000 (Wilmington, DE, USA). Finally, to identify the physical ends of the genomes, the purified fragments were sequenced at FIMM using a *fliC*-F4 primer that pointed towards the physical end of the phage genome. The ligation-PCR was carried out with phages fPS-7 and fPS-26. The primers used in this work (Oligomer Oy, Helsinki, Finland) are listed in Table 3.

### 2.6. Efficiency of Plating

Rough estimation of the efficiencies of plating (EOPs) of the phages for different host strains was carried out by spot titration. Briefly, a 90 µL/OD**_600_** volume of logarithmic phase host bacteria (OD**_600_**~0.8–1.0) grown in LB at RT was mixed with 3 mL of melted soft agar tempered to 50 °C. The soft agar was then poured onto an LA plate. After the solidification of the agar, 5 µL drops of serially 1:10-diluted phage stock were spotted on the soft agar. The dilutions up to 10^−8^ were made in SM buffer (50 mM Tris-HCl (pH 7.5), 0.1 M NaCl, 8 mM MgSO_4_ and 0.01% (*w*/*v*) gelatin). After the incubation, the plates were investigated for the presence of lysis. For each phage and host, the last dilution that showed lysis was selected and 50 µL of the selected dilution was mixed with the bacteria to the soft agar and plated to calculate the exact number of plaques. The EOPs of each phage with each host bacteria were calculated by dividing the phage titer on the target bacteria by the phage titer on the preferred bacterial host [40]. The experiment was carried out with two parallels and repeated at least twice.

### 2.7. Phage Inhibition Assay

The ability of lipopolysaccharides (LPS) to neutralize phages was tested using LPS purified from *Y. enterocolitica* O:3 strains 6471/76-c and its O-antigen deficient derivative YeO3-R1 (Table 1). The LPS samples were kindly provided by Dr. Katarzyna A. Duda, Borstel, Germany. Briefly, the LPS was dissolved in distilled water at 1 mg/mL and was then further ten-fold diluted up to 10^−4^. To 100 µL of these LPS preparations, 100 µL of fPS-7 suspension—containing about 27 plaque-forming units (PFUs)—was added, vortexed gently and incubated about 40 min at RT, and then mixed with the host bacteria (OD**_600_** ~0.3–1) and 3 mL of soft agar. The mixture was gently vortexed and plated onto LA plates. Plates having the phage and the indicator bacteria only were used as a control. After 24 h of incubation at RT, the formed plaques were counted. The experiment was carried out with two parallels and repeated at least twice.

### 2.8. One-Step Growth Curve Experiment

A one-step growth curve experiment was performed as described earlier [12,41,42] with modifications. Briefly, a mid-exponential phase culture of YeO:3-c was harvested by centrifugation and resuspended in 0.25 volume of fresh Tryptone Soya Broth (TSB). Phage (10^4^ PFU) was added to 1 mL of the bacterial culture and allowed to adsorb for 5 min. To remove the unadsorbed phages, the mixture was centrifuged and the pellet was resuspended in 1 mL of fresh TSB, from which a 100 µL aliquot was added to 9.9 mL of TSB (=tube A), from which further 1:10 (tube B) and 1:100 (tube C) dilutions were immediately prepared. The tubes were incubated shaking at RT. Forty microliters of sample were withdrawn from these tubes at 5–10-min intervals and plated in soft agar on LA plates with a 90 µL/OD**_600_** volume of the indicator bacteria (YeO:3-c). The experiments were performed at least twice with replicates.

### 2.9. Thermal, pH and Solvent Stability Tests

These experiments were conducted as described elsewhere [43,44,45] with modifications. To determine the thermal stability of the phages, 1 mL aliquots of phage (10^7^ PFU/mL) were incubated at 25, 30, 37, 45, 55, 65 and 75 °C for 1 h. After incubation, the phage titers were determined from serial 10-fold dilutions.

To estimate the effect of pH on phage stability, 100 µL of phage (10^5^ PFU/mL) was mixed with 900 µL of LB broth with different pH values. The pH range from 2 to 12 was adjusted by either 1 M HCl or 1 M NaOH. After 1 h incubation, the phage titers were determined from serial 10-fold dilutions. LB broth (pH 7.4) was used as a control.

The effect of chloroform and ethanol on phage stability was tested by mixing equal volumes of the phage (10^8^ PFU/mL) with chloroform or 80% ethanol (final concentration 40%). After 1 h incubation with intermittent shaking at RT, the phage titers in the supernatants were determined by the standard plaque assay. Phage mixed with SM buffer was used as a control.

### 2.10. Nucleotide Sequence Accession Numbers

The nucleotide sequences of the phage genomes were submitted to the European Nucleotide Archive (ENA) database. The accession numbers are listed in Table 2.

## 3. Results and Discussion

### 3.1. Phage Morphology

**Figure 2:**
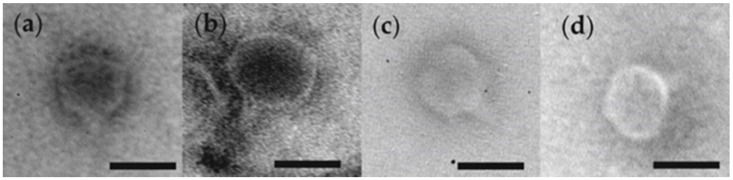
Transmission electron micrographs of fPS-21 (**a**), fPS-7 (**b**), fPS-52 (**c**) and fPS-50 (**d**). The bars indicate 50 nm.

All the tested phages had icosahedral heads of approximately 50 nm in diameter, and short tails of about 20–25 nm in length (Figure 2). Based on the morphological features, the fPS-phages belong to the order *Caudovirales* and the *Podoviridae* family [46].

### 3.2. Analysis of the fPS-phage Genomes

In this study, we continued to characterize the podoviruses isolated in our previous work [19]. While carrying out sequence and host range analyses of the phages we observed that we had not pure isolates of phages fPS-54 and fPS-55, instead we isolated from the fPS-54 stock the phage fPS-54-ocr with a completely different host range (see Section 3.3). As we did not obtain reliable sequence data for fPS-54 and fPS-55, we excluded them from this study. The 16 podoviruses included in this study are listed in Table 2. The genomes of all phages were linear double stranded DNA with size range of 38,391–40,451 bp and GC contents of 45.4–45.7 mol % (Table 2). The GC content was slightly lower than that of the host bacteria, 48.5 ± 1.5 mol % [47] and that of phage T7, 48.4% [13]. As is typical for T7 and other podoviruses [48], no tRNA genes were present in the fPS-phage genomes.

The fPS-phages encode their own RNAP and, similar to other T7-like phages, their RNAP and DNAP encoding genes map similarly [49]. This, in addition to the overall genomic sizes and predicted gene functions, placed the fPS-phages taxonomically as three new virus species under the *Autographivirinae* subfamily. In Table S1 the predicted gene products of all the fPS-phages were compared to corresponding products of fPS-7, and databases were searched using the gene products of fPS-7 for homologs, particularly from phages ϕYeO3-12, T3 and T7. Generally, there is a high level of similarity on the level of gene products within the same phage groups, and the similarity decreased when the proteins were compared between different phage groups.

#### 3.2.1. The Phylogenetic Position of the fPS-phages

**Figure 3:**
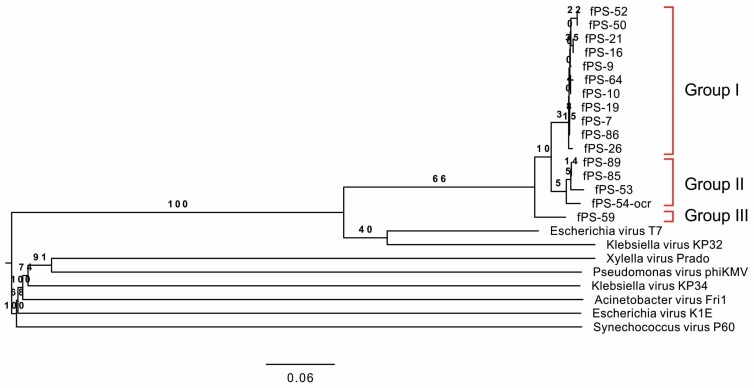
Phylogenetic tree generated by VICTOR using the complete genomic sequences of the fPS-phages and eight phage species, that were chosen to represent the eight genera of the *Autographivirinae* subfamily: *Escherichia* virus T7 (NC_001604.1), *Klebsiella* virus KP32 (NC_013647.1), *Xylella* virus Prado (NC_022987.1), *Pseudomonas* virus phiKMV (NC_005045.1), *Klebsiella* virus KP34 (NC_013649.2), *Acinetobacter* virus Fri1 (KR149290.1), *Escherichia* virus K1E (KY435490.1) and *Synechococcus* virus P60 (NC_003390.2). The genetic Groups I–III are indicated by red brackets.

The phylogenetic tree based on the complete genomic sequences of the 16 fPS-phages and eight *Autographivirinae* phages shows that the fPS-phages represent three related species (genetic groups) that are closest to the Escherichia virus T7 (Figure 3). Group I is the largest and includes 11 phages, Group II includes phages fPS-53, fPS-85, fPS-89 and fPS-54-ocr, and Group III contains only fPS-59. The nucleotide sequences of the fPS-phage genomes are highly identical (Figure S1), between 90 and 97% (Table S2). Based on the nucleotide sequence identities [50] between the genetic groups they represent three species with phages fPS-7, fPS-53 and fPS-59 as type species.

**Figure 4:**
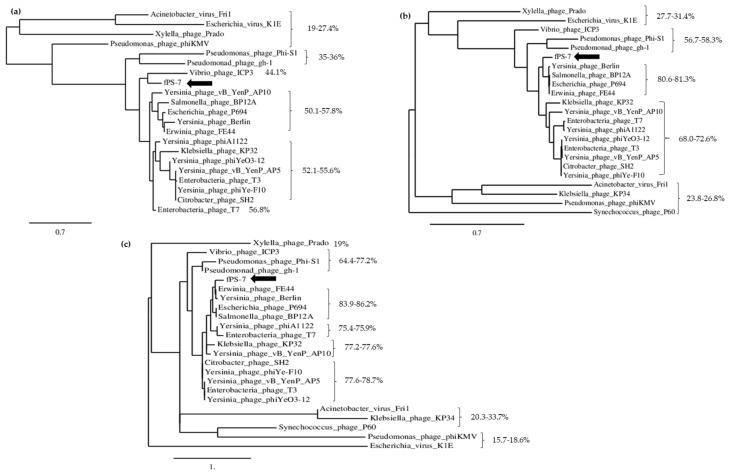
Phylogenetic tree analysis based on the alignments of amino acid sequences of DNA ligases (**a**), RNA polymerases (**b**) and capsid and scaffolding proteins (**c**) of *Autographivirinae* subfamily phages using BLASTP for the alignment and Phylogeny.fr for the phylogenetic tree construction. The black arrows indicate phage fPS-7. The identity-percentages of the three proteins of different phages to the proteins of fPS-7 are indicated.

Phylogenetic trees, constructed using the amino acid sequences of the DNA ligase, RNAP and the capsid proteins of fPS-7 and 22 different phages selected among the closest BLASTP hits, revealed, on the other hand, that the fPS-phages are most closely related to Yersinia phage Berlin, Erwinia phage FE44, Escherichia phage P694 and Salmonella phage PB12A (Figure 4). Of the eight *Autographivirinae* subfamily phages used in this tree, fPS-phages shared a close ancestor with Enterobacteria phage T7 and Klebsiella phage KP32. The fPS-phage identity-% of the three proteins to the studied phages ranged between 19–58% for the DNA ligase, 19–81% for RNAP and 19–86% for the capsid and scaffolding protein.

#### 3.2.2. The Genome Organization of the fPS-phages

**Figure 5:**
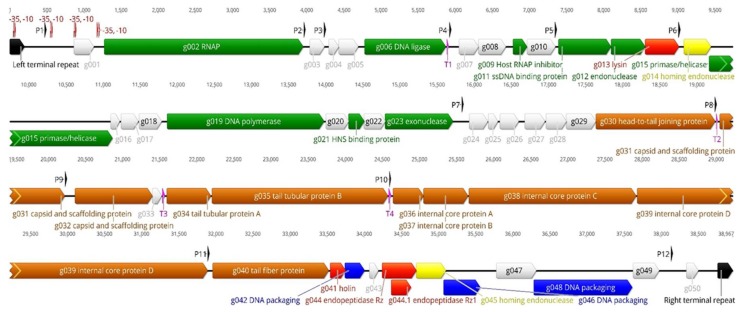
The genomic map of fPS-7. The predicted genes are arranged in the direction of transcription shown by different colored arrows. Genes involved in nucleotide metabolism, DNA replication, recombination or repair are shown in green. Genes involved in morphogenesis and virion structures are depicted in brown. Genes involved in DNA packaging and lysis, are shown in blue and red, respectively. Genes coding for hypothetical proteins or conserved phage proteins of unknown function are shown in light grey. Homing endonucleases are shown in yellow. Direct terminal repeats (DTRs) are shown in black. On top of the genome, the host RNA polymerase (RNAP)-dependent promoters are shown with red double-arrows labelled with −35 and −10, and the phage RNAP-dependent promoters with black arrows labelled from P1 to P12. Terminators are shown along the genome as purple triangles and labelled from T1 to T4. The genetic map was created using the Geneious software.

As a representative of the fPS-phages, the genomic map of phage fPS-7 is shown in Figure 5. The numbers of genes and proteins of the fPS-phages differ a little from each other due to deletions, replacements and duplications. To facilitate the presentation, we have described below the genes and gene products of phage fPS-7 (the Group I type species) as a representative of all phages. The unique genes of group II and III phages are described separately. It should be noted that all the functions of the predicted gene products are based on bioinformatic analyses and have not been confirmed experimentally. Apart from the hypothetical and phage proteins with unknown function, all the essential and conserved genes of T7-like phages were present in fPS-phages and most of them in the same order.

##### The Early Genes

The early genes of the phage genomes are likely comprised of genes *g001*–*g006* among which genes *g002* and *g006* encode the phage RNAP and the DNA ligase, respectively. In T7 phages, phage RNAP-encoded gene is transcribed by the host RNAP and the phage RNAP, is in turn responsible for the transcription of the remaining genes of the phage DNA. The host RNAP recognizes the host promoters found at the left end of the genome and starts transcription from them immediately upon the entry of the phage genome left end into the host cytoplasm [51]. Gp006, the DNA ligase, is 99–100% identical between the fPS-phages. In all the fPS-phages the early genes are highly conserved with only minor differences in intergenic regions.

The early gene predicted to encode a protein kinase was present only in Group II phages.

##### The Middle Genes

The gene *g009* encoding for host RNAP inhibitor is conserved and gene products are 100% identical in all the fPS-phages. There are two conserved genes upstream to *g009*, named *g007* and *g008*, coding for phage protein and hypothetical protein, respectively. However, the other middle genes encode for proteins involved in the DNA replication and repair, which include single stranded DNA-binding protein (Gp011), endonuclease (Gp012), primase/helicase (Gp015), DNA polymerase (Gp019) and exonuclease (Gp023). The detailed identity values of the amino acid sequences of these predicted genes are listed in Table S1.

##### The Late Genes

The predicted late genes encode proteins for prohead and tail fiber formation, DNA packaging proteins A and B, and host cell lysis. In T7, prohead formation is usually conducted by six proteins, that are all conserved in the fPS-phages, including Gp030, the phage collar/head-to-tail joining protein, Gp031 and Gp032, the capsid and scaffolding proteins, and Gp036–Gp039, the phage internal proteins [49]. It is unusual to see homing endonucleases in the late genes of T7-like phages, nevertheless, Gp045 appears to be such.

In T7, the tail tubular protein A, the tail tubular protein B and the phage tail fiber, play a crucial role in the specificity of the phage host range and in building up the viral tail [52]. Genes *g034*, *g035* and *g040* encoded tail tubular protein A, tail tubular protein B and phage tail fiber, respectively. While the N-terminal part of the tail fiber protein of the fPS-phages shows similarity to many tail fiber proteins in the databases, the C-terminus is unique (Figure S2); this explains the unique host specificities of the fPS-phages. Interestingly, HHpred detected a carbohydrate binding motif at the most C-terminal part of the fPS-phage tail fibers (Figure S2) supporting the observations that LPS functions as the surface receptor for the phages (see Section 3.3). Similar to T7, the late genes include genes encoding for the holin and the endopeptidases Rz and Rz1. The latter is encoded within a different frame in the Rz gene.

#### 3.2.3. Transcriptional Sequences

**Figure 6:**
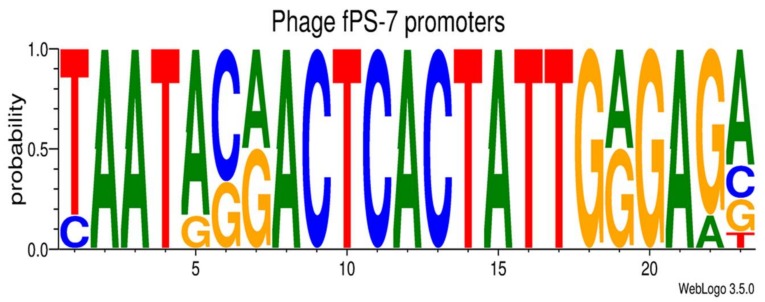
Sequence logo of fPS-7 promoters generated using the sequence logo generator [36] and the data in Table S3. The logo shows the residue probabilities at each position.

It is well known that the left end of T7-like phages genomes carry promoters used by the host RNAP upon the entry of the phage DNA into the host cytoplasm [53]. In the fPS-phages, three to six host RNAP promoters were identified by the BPROM program (Tables S3–S18). As expected, the identified promoters were all located upstream of *g002* encoding the phage RNAP. Altogether 11 to 13 putative phage RNAP specific 23 bp long promoter sequences were identified from the fPS-phage genomes (Tables S3–S18). The consensus sequences of the fPS-phage promoters were identical with the exception of fPS-54-ocr that exhibits a one-nucleotide difference. Moreover, the T7-consensus promoter sequence differs by only one nucleotide from that of fPS-phages. The predicted promoter consensus sequence of fPS-7 is shown in Figure 6.

Using the ARNold software, three to five transcriptional Rho-independent terminators were predicted from the fPS-genomes (Tables S3–S18). The identified putative terminators met the criteria of the Rho-independent transcriptional termination, i.e., a stretch of C and G sequence that forms the stem loop (hairpin) structure, followed by a stretch of Ts. The first Rho-independent terminator is located downstream the *g006*; at the end of the early region. The location of this terminator corresponds to the early terminator (TE) of T7 phages. The other intergenic predicted terminators are distributed within the middle and late genomic regions. There is a conserved terminator in all the fPS-phages which lies between the gene encoding collar protein and capsid protein, respectively. The location of this terminator may correspond to Tø in T7 phages, which is regarded as a strong terminator [53].

#### 3.2.4. The Terminal Repeats (TR)

The results revealed that the fPS-phage genomes carry direct terminal repeats (DTRs) with lengths ranging between 190 and 224 bp. The DTRs are non-coding DNA sequences that are found on both ends of some phage genomes [54]. During DNA replication, TRs overlap, allowing the formation of long concatemer composed of the replicated DNA linked end to end by the shared TRs [55]. In T3 and T7 phages, the duplication of the TRs during DNA packaging is crucial, otherwise half of the DNA concatemers will not be packaged [56]. In all the fPS-phage genomes, the TRs were highly similar (Figure S3), demonstrating some differences in the form of deletions and duplications within the repeats (Figure S3, boxes 1 and 2). In addition, within the TRs, three variable-length poly-C stretches are present.

#### 3.2.5. Microevolution of the fPS-phages

The multiple alignment of the genomic DNA sequences (Figure S1) illustrate the differences between the fPS-phage genomes. Most interesting differences at nucleotide level are summarized in Table 4 and described and illustrated in details in the Supplementary data. In general, the genomes showed the greatest variability close to both TRs.

### 3.3. Characterization of the Phage Receptors

Many Yersinia phages use LPS as a receptor [12,16,57,58,59]. To test whether this was also the case with the fPS-phages we analyzed the sensitivity of a set of *Y. enterocolitica* serotype O:3 LPS mutants (Table 1) to the phages. LPS, a component found on the outer membrane of Gram-negative bacteria, is in *Y. enterocolitica* O:3 composed of lipid A (LA), inner core (IC), outer core (OC), and O-antigen (O-ag) [60]. The EOP values of the fPS-phages were determined on the *Y. enterocolitica* O:3 LPS mutants (Table 5).

The results demonstrated that different fPS-phages had different EOP values on different LPS mutants. With the exception of fPS-53 and fPS-54-ocr, all the tested phages preferentially infected the wild type strain YeO3; for these, the EOP was set to one. On the other hand, most of the phages exhibited low EOPs, or did not infect at all YeO3-R1; the O-ag lacking strain. Based on this we speculate that O-ag may function as a receptor for these phages. The OC could be the receptor for fPS-53, fPS-85 and fPS-89; as they were unable to infect the OC lacking strains.

While carrying out these experiments, we noticed that one stock of fPS-54 was able to infect the YeO3-c-OCR strain. Closer analysis of the stock revealed that it contained two different phages, the one originally identified as fPS-54, and a second one that we named as fPS-54-ocr. We speculate that this was possibly due to appearance of spontaneous fPS-54-resistant mutants (i.e., lacking both O-ag and OC) into the culture. These O-ag/OC-mutants would have sustained the growth of the fPS-54-ocr phages, albeit to a lower titer in the stock. To identify whether the LPS IC would be the receptor of fPS-54-ocr, three deep-rough LPS mutants YeO3-R1-M205, YeO3-R1-M164 and YeO3-R1-M196 with different levels of truncations in the IC (Table 1) were tested with the phage. Of the mutants YeO3-R1-M205 has only the Kdo-residues of the IC present, and as it, and the two other mutants with less truncated IC [22], were all successfully infected by fPS-54-ocr, we conclude that the phage does not use the IC of LPS as a receptor. Moreover, fPS-54-ocr was able to infect YeO3-c-OCR-ECA strain which lacks the ECA (Enterobacterial Common Antigen).

In adsorption experiments, the fPS-phages exhibited only minor adsorption (20–40%) during the first 5 min to their host cells and this was not much increased even if the adsorption time was extended up to 60 min. This is reminiscent of the low adsorption rates of *Pseudomonas aeruginosa* phages LKD16, φKMV and φS1 [61].

#### The Phage fPS-7 Uses the O-ag as a Receptor

**Figure 7:**
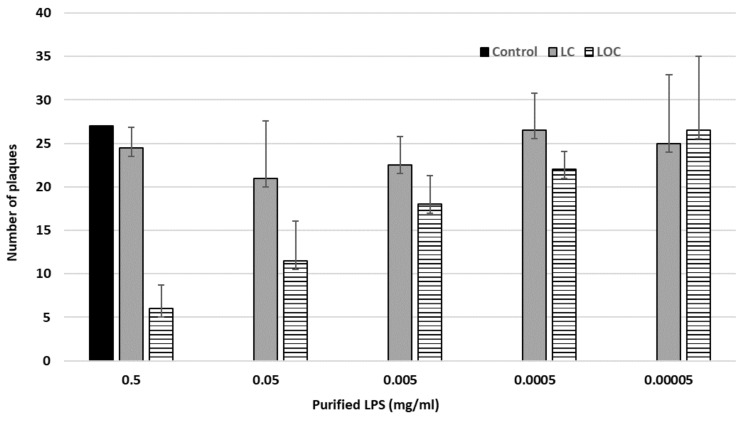
Inhibition assay of the phage fPS-7 with the purified LPS derived from wild type Ye O:3 strain 6471/76 and from the rough derivative YeO3-R1. The values shown represent the average of two independent experiments. The black bars (the control) represent the number of plaques shown when plating fPS-7 on the host bacteria without addition of lipopolysaccharide (LPS). The grey bars (LC) represent the number of plaques appeared when plating the mixture of fPS-7 with the purified LC on the permissive host, which showed an average number of plaques of 21 to 26.5 (relative to the host which was around 28). A clear drop in the number of plaques is shown on the LOC bars; the least number of plaques is seen with the undiluted LOC preparation and the dose response is demonstrated upon the dilution of the LOC. Error bars represent the standard deviations of the means of number of plaques obtained from the phage assay.

The EOP results of the LPS mutants suggested that some phages of the Group I use O-ag as a receptor in the process to infect the host. To verify this, we carried out inhibition experiments with fPS-7, one of the group I phages, by using purified LPS isolated from two different YeO3 strains, i.e., the wild type strain 6471/76-c with LPS containing LA, O-ag and core (both IC and OC) and the rough strain YeO3-R1 that lacks the O-ag. We abbreviate these LPSs as LOC (L: lipid A, O: O-ag and C for the core; wild type), and LC (rough), respectively. The results, presented in Figure 7, demonstrated that the LOC but not the LC-type LPS was able to neutralize the phage, providing convincing evidence that the O-ag is used by fPS-7 as a receptor.

### 3.4. One-Step Growth Curves

For these experiments we selected phages fPS-9, fPS-89 and fPS-59 to represent the genetic groups I, II and III, respectively. One-step growth curves of these phages were very similar and the curves of fPS-9 and fPS-59 are shown in Figure S4. The latent periods of the phages were 30–35 min and the burst sizes were 170–200 PFU per infected cell.

### 3.5. Thermal, pH and Solvent Stability of the Phages

Knowledge of phage viability and stability is important for phages to be used as biocontrol agents. In this respect the thermal, pH and solvent stability of fPS-9, fPS-89 and fPS-59 was tested. Generally, the phages were stable up to 37 °C, however, at 45 °C the phage counts were already significantly reduced (25–46% survival), and at 55 °C only <1% of the phages retained infectivity. At 65 and 75 °C practically all phages were inactivated (Figure S5). The phages were highly stable at pH 7.4 and 9, while at pH 12 there was a significant drop in the survival-% of the phages. The phages did not tolerate the acidic media at pH 2 and 4 (Figure S6). While the phages tolerated well the chloroform treatment, they were completely inactivated by 40% ethanol.

## 4. Conclusions

In this study, sixteen Yersinia phages isolated from different Finnish pig farms were characterized. The nucleotide sequences of the genomes revealed that the phages were closely related and shared significant similarity, even though they were isolated from different pig farms. The genome size and organization as well as the predicted gene functions are highly similar to T7 phages. In addition, as the fPS-phages encode their own RNAP gene, they can be classified within the *Autographivirinae* subfamily, in the genus *T7virus.* Different parts of the host LPS were demonstrated to function as receptor for all the phages except for fPS-54-ocr that probably uses an outer membrane protein as a receptor. Finally, as the fPS-phages are lytic and devoid of any harmful genes they would be safe to use in phage therapy in the future.

## Figures and Tables

**Table 1:** Bacterial strains used in this work.

Bacterial Strain	Comments	Reference/Source
6471/76 (YeO3)	Serotype O:3, wild type. Human stool isolate	[20]
6471/76-c (YeO3-c)	Virulence plasmid-cured derivative of 6471/76	[20]
YeO3-R1	Spontaneous rough derivative of YeO3-c	[21]
YeO3-R1-M164	*waaF::Cat-Mu*. derivative of YeO3-R1. Clm^R^	[22]
YeO3-R1-M196	*galU::Cat-Mu* derivative of YeO3-R1. Clm^R^	[22]
YeO3-R1-M205	*hldE::Cat-Mu* derivative of YeO3-R1. Clm^R^	[22]
YeO3-c-OC	∆(*wzx-wbcQ*), derivative of 6471/76-c, a virulence plasmid cured derivative of 6471/76	[23]
YeO3-c-OCR	Spontaneous rough derivative of YeO3-c-OC	[23]
YeO3-c-OCR-ECA	Δ(*wzx-wbcQ*) Δ(*wzzE–wzyE*). OPS-, outer core- and ECA-negative derivative of 6471/76-c, Km^R^	[24]

**Table 2:** Characteristics of the *Y. enterocolitica* specific fPS-phages.

Phage	Farm	Genome Size (bp)	GC Content (%)	ORFs (*n*)	Terminal Repeat (bp)	Promoters (*n*)	Terminators (*n*)	Accession Number
fPS-7	3	38,966	45.6	51	200	12	4	LT961840
fPS-9	3	39,034	45.6	52	200	13	5	LT960606
fPS-10	3	39,179	45.5	51	202	12	4	LT962907
fPS-16	3	39,227	45.5	51	200	12	4	LT962906
fPS-19	3	38,938	45.6	51	200	12	4	LT961838
fPS-21	3	39,180	45.5	51	202	12	4	LT961844
fPS-26	5	38,792	45.7	51	205	12	4	LT961836
fPS-50	7	39,764	45.5	50	224	12	4	LT961843
fPS-52	7	39,888	45.4	50	224	12	4	LT961837
fPS-53	7	40,451	45.4	50	196	11	3	LT962379
fPS-54-ocr	7	40,074	45.5	49	200	11	4	LT962475
fPS-59	21	38,391	45.7	47	190	11	4	LT961845
fPS-64	5	39,326	45.5	50	204	12	4	LT961846
fPS-85	26	40,429	45.4	50	196	11	3	LT962380
fPS-86	28	39,024	45.6	51	215	12	4	LT961842
fPS-89	25	40,405	45.4	50	195	11	3	LT961841

**Table 3:** The primers used in this work.

Primer	Primer Sequence (5′-3′)
fPS-7-F	CCATAGGCCCTCTCAGTCAT
fPS-7-R	CAACCTCGTGATGTCTTACCG
*flic*-F3	TCAACCATCACCAACCTGAA
*flic*-R3	TCTTTTGCGCTGTTGATACG
*flic*-F4	GGATGAGCCTGCCGATAATA

**Table 4:** Microevolution of the fPS-phages. Summary of the genome differences between fPS-phages. The Box numbers refer to Figure S3, where the differences are indicated by numbered boxes.

Box	Description of Differences	Consequence
1	10 bp repeats	Differences in the length of left terminal repeat (TR) (also valid for right TR)
2	12 bp repeats & poly-C tracks	Differences in the length of left TR (also valid for right TR)
3	Between 11 and 34 repeats of different variations (Table S19)	Different distances between the left TR and phage promoter P1
4	3–5 repeats of 28 bp	Variation in Group Ib phages on promoter P1 left flanking regions
5	4–5 repeats of 23 bp	Variation in Group Ib phages on promoter P1 right flanking regions
6	1380 bp insertion	Gene *g002* of Group II that is absent from Group I and III phages
7	274 bp insertion in Group Ia, and 274 + 342 (=616 bp) insertion in Group Ib genomes	Two variants of *g003* in Group Ia and Ib genomes. The gene is absent from Groups II and III
8	303 bp insertion	*g012* of fPS-59, absent in Group I and II
9	94 bp insertion	May encode a 30 amino acid long polypeptide in fPS-59
10	Poly-G_7_ to G_13_ stretch	Part of ribosomal binding site (TAAGG)
11	422 bp insertion	Extra gene in Group II and III phages
12	136 bp fragment replacing a 127 bp fragment	The *g022* in Group II phages has different 5′-end resulting in different N-terminal sequence of 15 amino acids.
13	140 bp region	A pseudogene in Groups Ib, II and III corresponding to Group Ia gene *g027*
14	6 or 21 bp deletions and short duplications within a 40 bp GC-rich stretch	In frame deletions and substitutions in Group Ia gene *g028* homologs in corresponding Groups Ib, II and III genes
15 & 16	386 bp deletion	The Group I gene *g029* is missing from both fPS-54-ocr and fPS-59
17	Poly-T_7_ to T_9_ stretch	Downstream of Rho-independent terminator
18	Variable region	3′-thirds of the genes encoding tail fiber protein in fPS-54-ocr and fPS-59 are highly divergent from the others receptor binding domains
19	207 bp deletion	Group I *g043* is truncated and fPS-59 lacks the gene
20	23 bp duplication	Alters the 3′-end frame of the Group II phage gene thereby altering the last eight codons
21	1–5 copies of an 80 bp repeat	Noncoding region downstream of phage promoter P12
22	Five different repeat sequences of 10–22 bp in size	Variability in length of the right TR flanking region (see also Table S20)

**Table 5:** The EOP of the phages on *Y. enterocolitica* serotype O:3 LPS mutants. The EOP on the preferred host was set to 1.

Phage	Strains and LPS Compositions			
	YeO3	YeO3-R1	YeO3-c-OC	YeO3-c-OCR
	LA-IC-OC-Oag	LA-IC-OC	LA-IC-Oag	LA-IC
fPS-7	1	0	0.2 × 10^−2^	0
fPS-9	1	1.2 × 10^−3^	0.8 × 10^−3^	0
fPS-10	1	3.3 × 10^−5^	5 × 10^−3^	0
fPS-16	1	0	0.5 × 10^−2^	0
fPS-19	1	2 × 10^−4^	9.5 × 10^−5^	0
fPS-21	1	0	1.2 × 10^−4^	0
fPS-26	1	0	0.7 × 10^−3^	0
fPS-50	1	0.3 × 10^−4^	0.8 × 10^−3^	0
fPS-52	1	0	0	0
fPS-53	0.5	1	0	0
fPS-54-ocr	0	0	0	1
fPS-59	1	0	1.7 × 10^−1^	0
fPS-64	1	0.6 × 10^−6^	1.3 × 10^−3^	0
fPS-85	1	0.5	0	0
fPS-86	1	0.1 × 10^−4^	0.5 × 10^−2^	0
fPS-89	1	0.6 × 10^−2^	0	0

## Supplementary Materials

The following are available online at http://www.mdpi.com/1999-4915/10/4/174/s1. Figure S1: Overview of the multiple alignment of the fPS-phage genomes, Figure S2: Multiple alignments of the phage tail fiber amino acid sequences, Figure S3: Multiple alignment of genomic nucleotide sequences of the fPS-phages, Figure S4: One-step growth curves of phages fPS-9 and fPS-59, Figure S5: Thermal stability of phages fPS-9, fPS-59 and fPS-89, Figure S6: The effect of pH on the stability of phages fPS-9, fPS-59 and fPS-89, Table S1: Comparison of the fPS-phage gene products to those of fPS-7, Table S2: Whole genome DNA sequence comparisons of fPS-phages using the EMBOSS stretcher program, Tables S3–S18: Predicted promoters and terminators, Table S19: Repeats in the fPS-phage genomes between left TR and phage promoter P1, Table S20: Repeats in the fPS-phage genomes between right TR and phage promoter P12.
