# Supplementary material for: Genomic Characterization of Sixteen Yersinia enterocolitica-Infecting Podoviruses of Pig Origin
Source: Viruses. 2018 Apr 3;10(4):174. doi: 10.3390/v10040174 (PMC5923468; doi:10.3390/v10040174)
Supplement: Supplementary file 1 [file viruses-10-00174-s001.zip › Salem_SI_rev.pdf]

viruses

MDPI

Supplementary material

Genomic Characterization of Sixteen *Yersinia enterocolitica* –infecting Podoviruses of Pig Origin

Mabruka Salem [1,2], Mikael Skurnik [1,3,*]

1 Department of Bacteriology and Immunology, Medicum, Research Programs Unit, Immunobiology, University of Helsinki, Helsinki, Finland
2 Department of Microbiology, Faculty of Medicine, University of Benghazi, Benghazi, Libya; Mabruka.salem@helsinki.fi
3 Division of Clinical Microbiology, Helsinki University Hospital, HUSLAB, Helsinki, Finland; mikael.skurnik@helsinki.fi
* Correspondence: mikael.skurnik@helsinki.fi; Tel.: +358-2941-26464

1. Comparison of the fPS-phage genomes

The nucleotide sequences of the 16 phage genomes show a high level of sequence similarity (between 90–97%) (Figure S1, Table S1). Overview of the fPS-genomes is shown in Figure S1 below and detailed description of the differences in Figure S2. As a representative of the fPS-phages, the genomic map of phage fPS-7

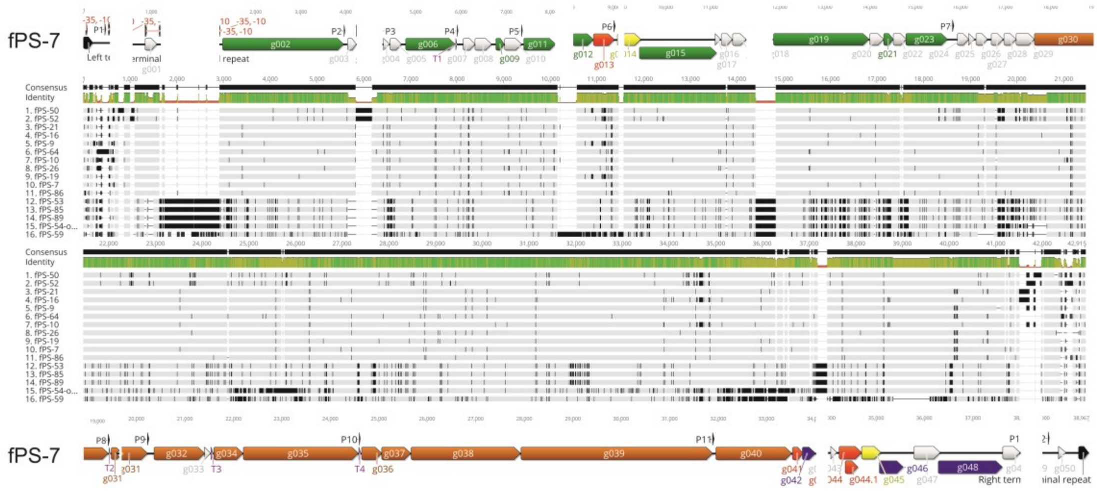

Figure S1. Overview of the alignment of the fPS-genomes. The genomes were aligned using the MAFFT program and the clustal alignment was imported to Geneious. The genomes are represented by grey bars within which the differences from the consensus sequence are indicated by black vertical lines. The consensus sequence and identity graph are given below the scale line. The genetic map of the genome of fPS-7 is shown aligned to the multiple alignment to indicate the genes located in the conserved regions.

A. Clustal W multiple sequence alignment of tailfiber sequences of fPS-7 and related podoviruses

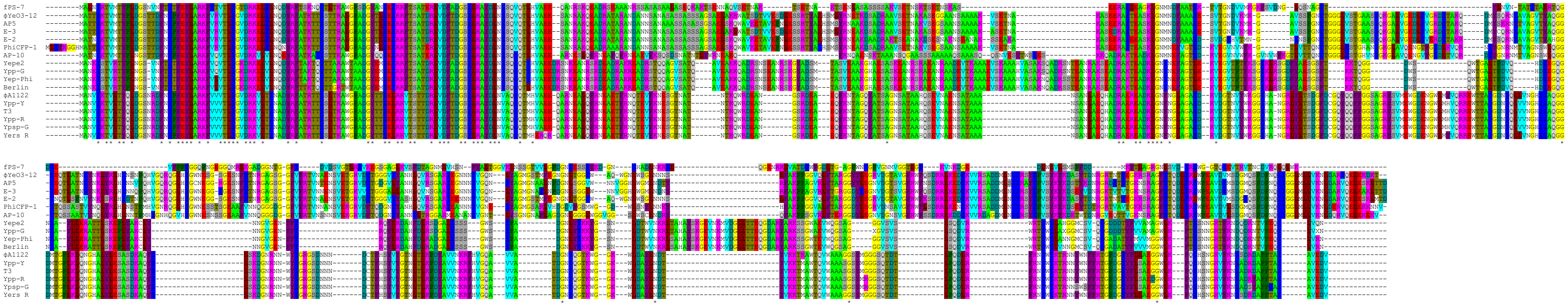

Accession numbers for the aligned sequences: fPS-7, ; ϕYeO3-12 np_052117.1; AP5, yp_009102835.1; E-3, yp_009198336.1; E-2, aka61571.1; PhiCFP-1, yp_009205690.1; AP-10, yp_009187309.1; Yepe2, yp_002003351.1; Ypp-G, afk13480.1; Yep-Phi, yp_009014859.1; Berlin, yp_919019.1; ϕA1122, np_848304.1; Ypp-Y, afk13385.1; T3, np_523342.1; Ypp-R, afk13438.1; Ypsp-G, afk13534.1; Yers R, agb07363.1

B. Clustal W multiple sequence alignment of tailfiber sequences of fPS-phages

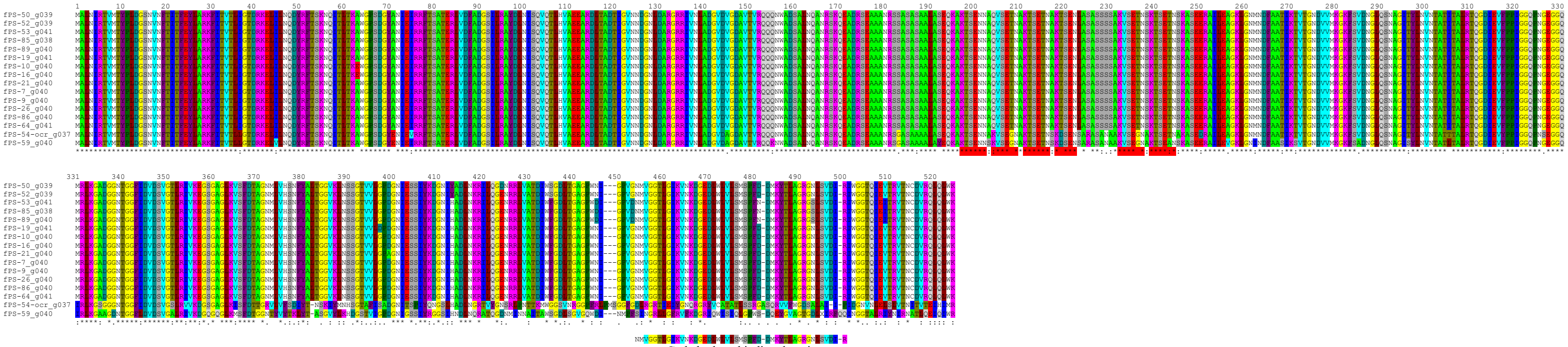

Carbohydrate binding domain

Figure S2: Multiple alignments of the phage tail fiber sequences.

2. Microevolution details of the fPS-phages

The sequence alignment revealed interesting differences between the nucleotide sequences of the fPS-phages. These are illustrated in more details in Figure S2. To help to find the locations in Figure S2 discussed below we have used boxes where the relevant alignments are framed and labelled as boxes 1-22. These are summarized in the main text, section 3.2.5 (Table 4).

The genomes showed great variability close to the TRs. For example, there are different numbers of repeats present in both TRs, those of left TR are indicated in **Boxes 1 and 2** (Figure S2). The Group III phage fPS-59 carries a putative gene (*g001*) that starts at the end of left TR; this gene is absent from all other phages. Flanking the the left TR, before phage promoter P1, different numbers of two repeat variations of 10 or 11 bp are present ranging in total between 11 and 34 repeats (Table S19, Figure S2, **Box** 3). While the phage promoter P1 region is highly conserved in all phages, the Group Ib phages carry on both flanking regions 1─5 repeats of 28 and 23 bp, respectively (**Boxes 4 and 5**).

The predicted gene *g001* of Group I differs from that of groups II and III. The latter two miss the 5′-region of *g001* present in Group I phages and the 3′-ends also differ (**Box 6**). Thereafter, the Group II phages carry an extra gene (annotated as *g002* in them) that is absent from Group I and III phages. The gene encodes for a predicted protein kinase. The Group I *g002*, and Group II and III *g003*, encode for phage RNAP. While the latter gene is overall highly identical; the first 5′-end 150 bp of Group II gene present differences when compared to Group I and III (Figure S2).

Immediately after the RNAP encoding gene *g002*, Groups Ia and Ib have *g003* that is longer in Group Ib due to an insertion of 342 bp fragment in fPS-50 and fPS-52 genomes (Box 7). That causes a frame shift and generates into Group Ib *g004* that shares most of its sequence with the 3′-end of Group Ia *g003*. The Group Ia *g003* sequence is absent from groups II and III phages. The *g004*─*g011* block of genes is conserved in all fPS-phages. Thereafter only the sequence of fPS-59 differs from those of Group I and II sequences in such that it has insertions between the Group I genes *g011* and *g012* (**Box 8**) and between *g013* and *g014* (**Box 9**). The first insertion in fPS-59 contains the predicted gene *g012* that ends overlapping with a few codons the following endonuclease coding gene that differs from the Group I and II gene showing 72% nucleotide and 76% amino acid sequence identity. Most differences were located in the 5′-end of the endonuclease coding gene. The second insertion in fPS-59 is 94 bp long and may encode a 30 amino acid long polypeptide (**Box 9**).

Thereafter the genomes are almost identical over the genes *g014*─*g018*. Between genes *g016* and *g017* apparently forming part of ribosomal binding site (TAAGG) is a poly-G stretch that varies between the Group I phages from G7─G13 (**Box 10**). In Group II and III phages it is replaced by GGAG.

Sharply before the RBS of the DNA polymerase encoding gene (*g019*), Group II and III phages have a 422 bp insertion (**Box 11**) that contains an extra gene (Group II *g019* and fPS-59 *g020*). The *g019*─*g024* genes are conserved in all phages except for the *g022* that in Group II phages has different 5′-end resulting in different N-terminal sequence of 15 amino acids (**Box 12**). The Group I genes *g025* and *g026* are missing from fPS-59.

An ORF corresponding to Group Ia gene *g027* is present in Groups Ib, II and III but has lost its start codon forming a pseudogene. The start codon in *g027* is UUG preceded by a perfect RBS (TAAGGAGG) that is conserved in all phages, however, on both sides of the RBS the Group Ia sequence differs from that of Group Ib, II and III sequences over a stretch of 116 bp. This has generated the pseudogene in the Groups Ib, II and III (**Box 13**). The next variation is found within the Group Ia gene *g028*: in the corresponding gene of groups Ib, II and III there are 6 or 21 bp deletions and short duplications within a 40 bp GC-rich stretch (**Box 14**).

The Group I gene *g029* is missing from both fPS-54-ocr and fPS-59, and from the sequence data it looks like that the Group I gene *g029* has been inserted into this locus and has replaced the RBS and its immediate upstream AT-rich sequence present in fPS-54-ocr and fPS-59 (**Boxes 15 and 16**).

All the phage genomes carry almost identical genes corresponding to Group I genes from *g030* to 5′-terminus of *g035*. These genes encode for structural proteins. Between *g033* and *g034* RBS is a poly-T stretch ranging from T_7 to T_9 that is part of the Rho-independent terminator (**Box 17**). The *g035*

is predicted to encode for tail tubular protein B. The corresponding gene of fPS-54-ocr and fPS-59 differs from that of the others with ca 90% identity to them.

Genes *g036*—*g039* encoding for structural proteins are conserved in all phages. Also the 5′-third of *g040* encoding the receptor binding tail fiber protein is conserved, however, the 3′-thirds of the genes in fPS-54-ocr and fPS-59 are highly divergent to the others and also to each other, reflecting their different host ranges (**Box 18**).

All the phage genomes carry almost identical genes corresponding to Group I genes *g041*—*g050* with some exceptions. The Group I phages differ from Group II and III phages at *g043*. While fPS-59 lacks the gene; the Group II phages have an insertion within the *g043* and this causes that the Group I and II genes have different 3′-ends (**Box 19**). The *g047* is missing from fPS-59 due to a sharp deletion: the sequences on both sides of the deletion are almost 100% identical. In fPS-9, a deletion of 86 bp causes truncation of the Group I *g049* gene, while in Group II phages there is a 23 bp duplication that alters the frame in the 3′-end of the gene thereby altering the last eight codons (**Box 20**). Downstream of phage promoter P12, an 80 bp repeat was present in 3—5 copies in fPS-50, fPS-52, fPS-21, and fPS-16, while in all other phages it was present as one copy (**Box 21**).

Analogous to the region flanking left TR, also the region upstream the right TR the phages varied and contained variable numbers of five different repeat sequences of 10—22 bp in size (Table S20, Figure S2, **Box 22**).

Figure S3. The MAFFT generated multiple alignment of fPS-phage genome sequences from Figure S1 at nucleotide sequence level. The bases are highlighted in different colors to facilitate comparison. On top of the alignments are indicated the locations of the terminal repeats, -35 and -10 boxes of host RNAP-specific promoters, the phage RNAP-specific promoters (P1 – P12) and genes. The boxes 1 – 22 locate the differences discussed in the text.

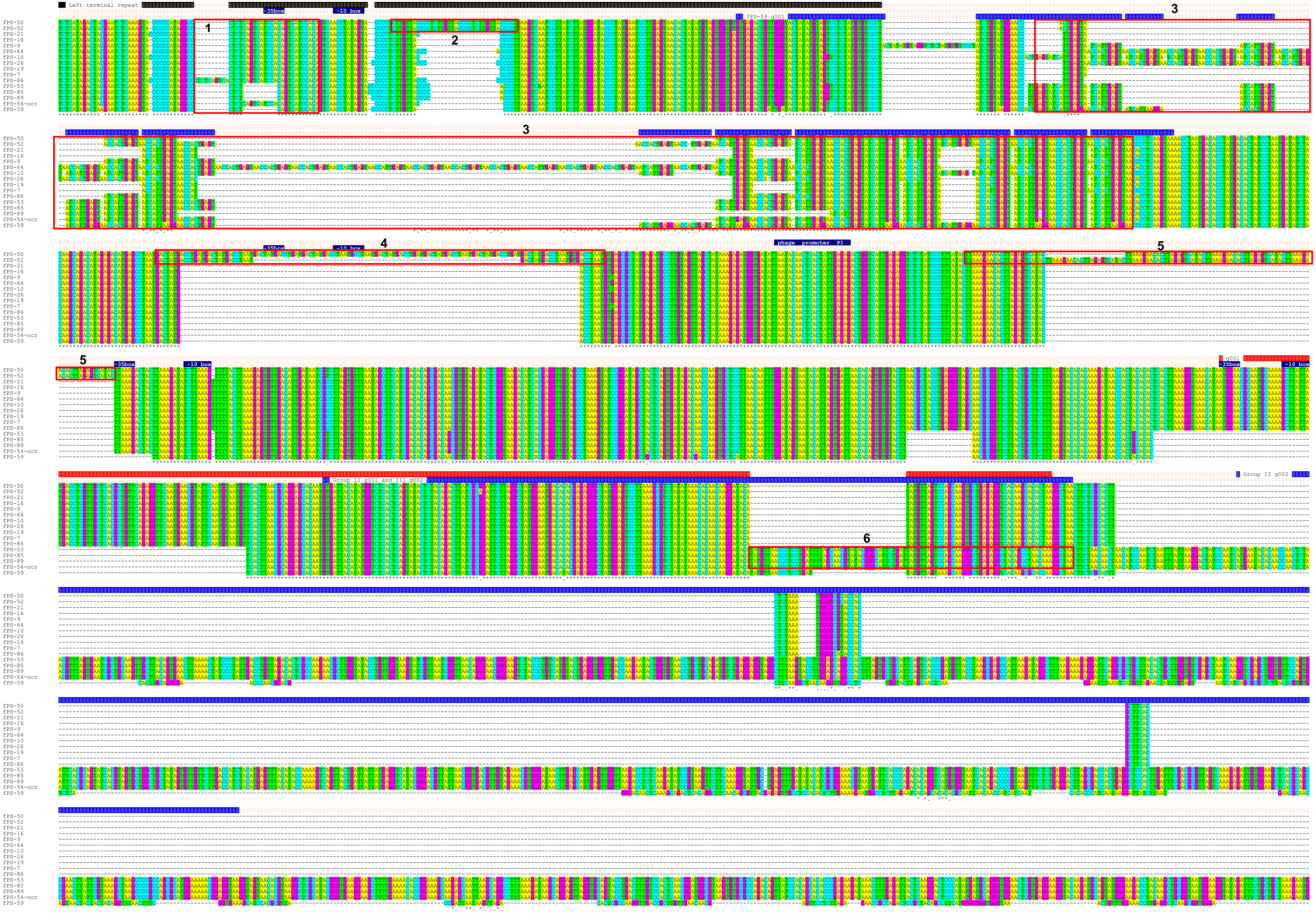

Left terminal repeat
-35box
-10 box
fPS-59 g001
phage promoter P1
Group II g001 and III g002
Group II g002
g001
1
2
3
4
5
6
fPS-50
fPS-52
fPS-21
fPS-16
fPS-9
fPS-64
fPS-10
fPS-26
fPS-19
fPS-7
fPS-86
fPS-53
fPS-85
fPS-89
fPS-54-ocr
fPS-59

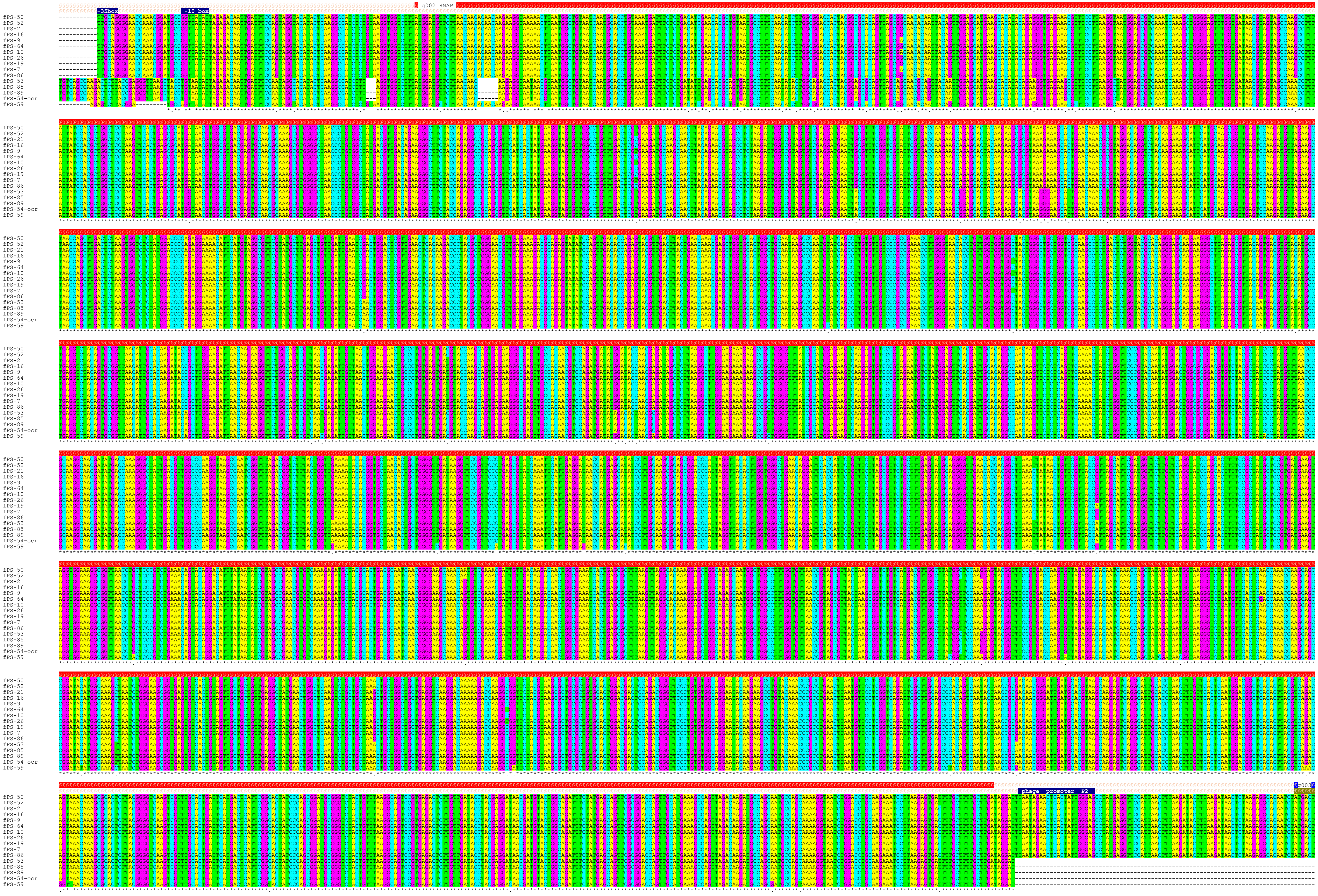
-35box
-10 box
g002 RNAP
phage promoter P2
g003
fPS-50
fPS-52
fPS-21
fPS-16
fPS-9
fPS-64
fPS-10
fPS-26
fPS-19
fPS-7
fPS-86
fPS-53
fPS-85
fPS-89
fPS-54-ocr
fPS-59

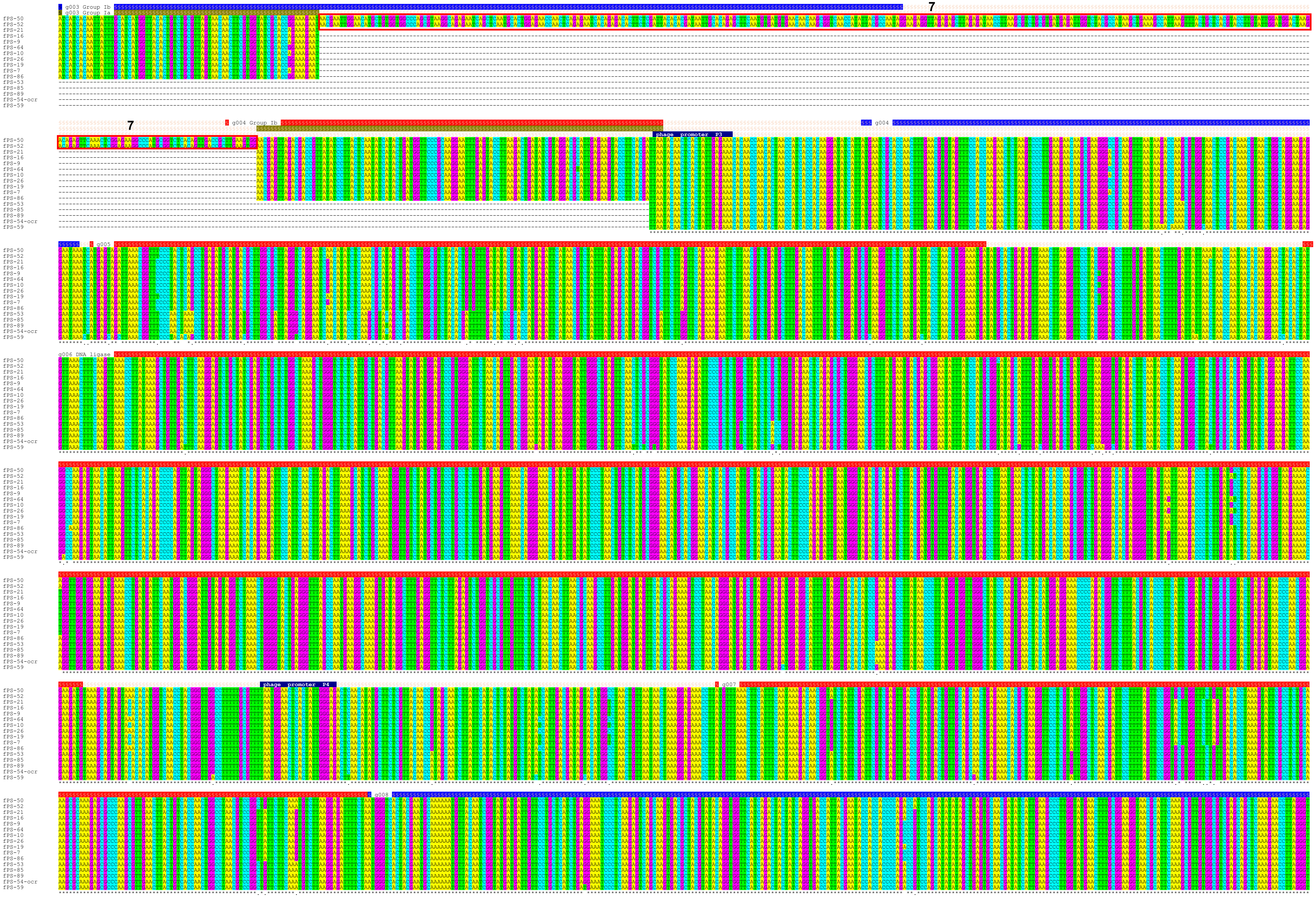
g003 Group Ib
g003 Group Ia
7
fPS-50
fPS-52
fPS-21
fPS-16
fPS-9
fPS-64
fPS-10
fPS-26
fPS-19
fPS-7
fPS-86
fPS-53
fPS-85
fPS-89
fPS-54-ocr
fPS-59
g004 Group Ib
g004
phage promoter P3
g005
g006 DNA ligase
phage promoter P4
g007
g008

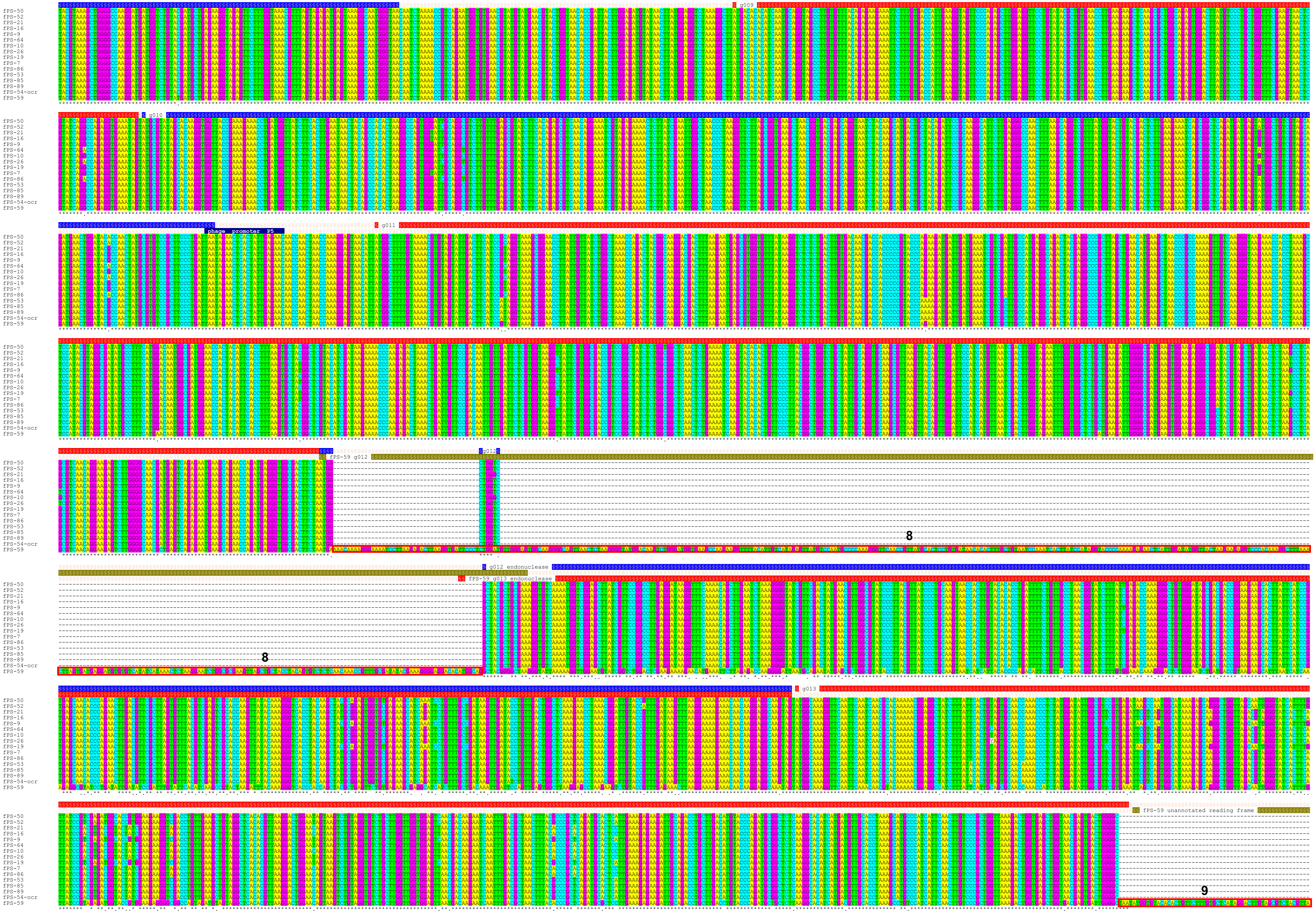

fPS-50
fPS-52
fPS-21
fPS-16
fPS-9
fPS-64
fPS-10
fPS-26
fPS-19
fPS-7
fPS-86
fPS-53
fPS-85
fPS-89
fPS-54-ocr
fPS-59
g009
g010
phage promoter P5
g011
fPS-59 g012
g012
8
g012 endonuclease
fPS-59 g013 endonuclease
g013
fPS-59 unannotated reading frame
9

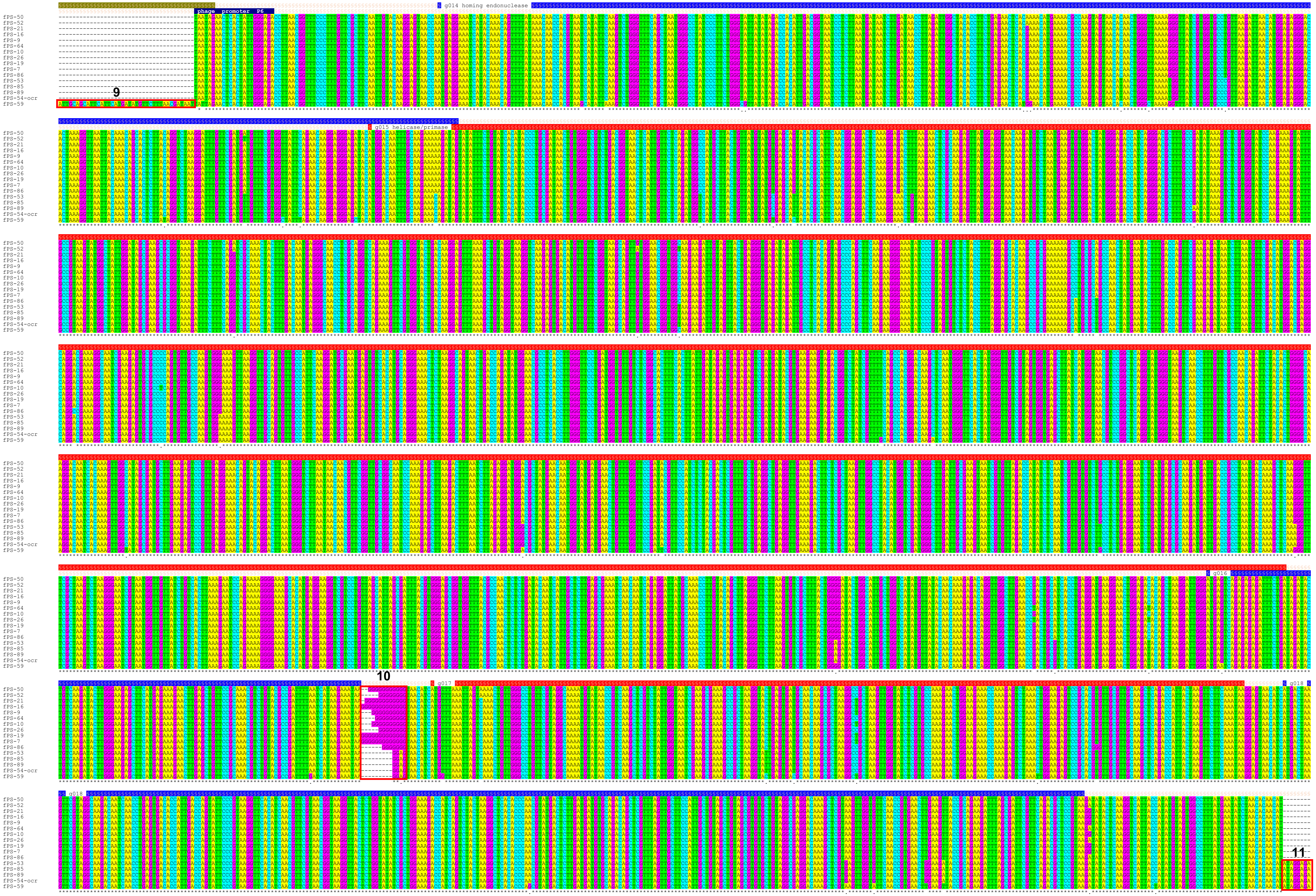
g014 homing endonuclease
phage promoter P6
g015 helicase/primase
g016
g017
g018
9
10
11
fPS-50
fPS-52
fPS-21
fPS-16
fPS-9
fPS-64
fPS-10
fPS-26
fPS-19
fPS-7
fPS-86
fPS-53
fPS-85
fPS-89
fPS-54-ocr
fPS-59

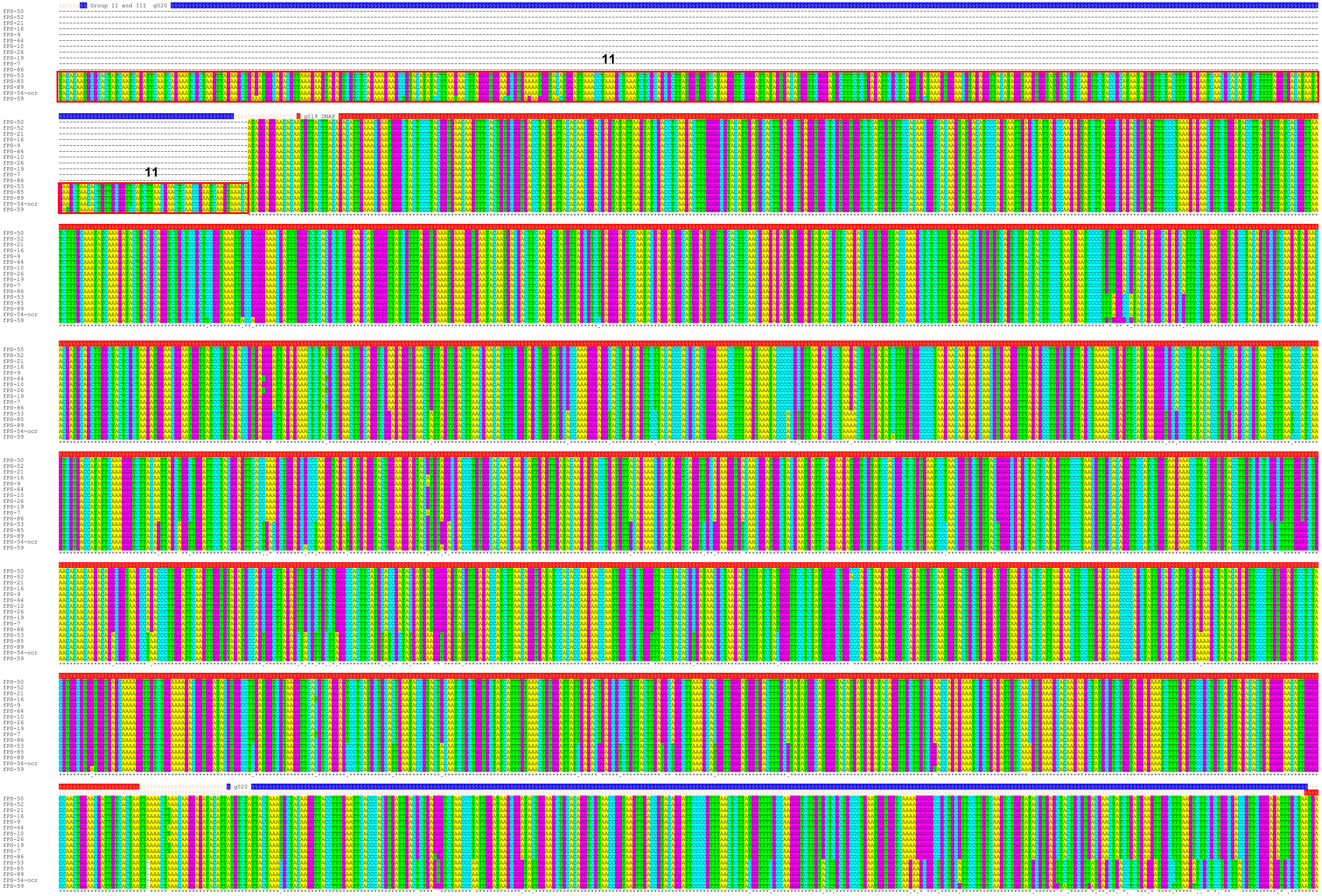
Group II and III g020
11
g019 DNAP
g020
fPS-50
fPS-52
fPS-21
fPS-16
fPS-9
fPS-64
fPS-10
fPS-26
fPS-19
fPS-7
fPS-86
fPS-53
fPS-85
fPS-89
fPS-54-ocr
fPS-59

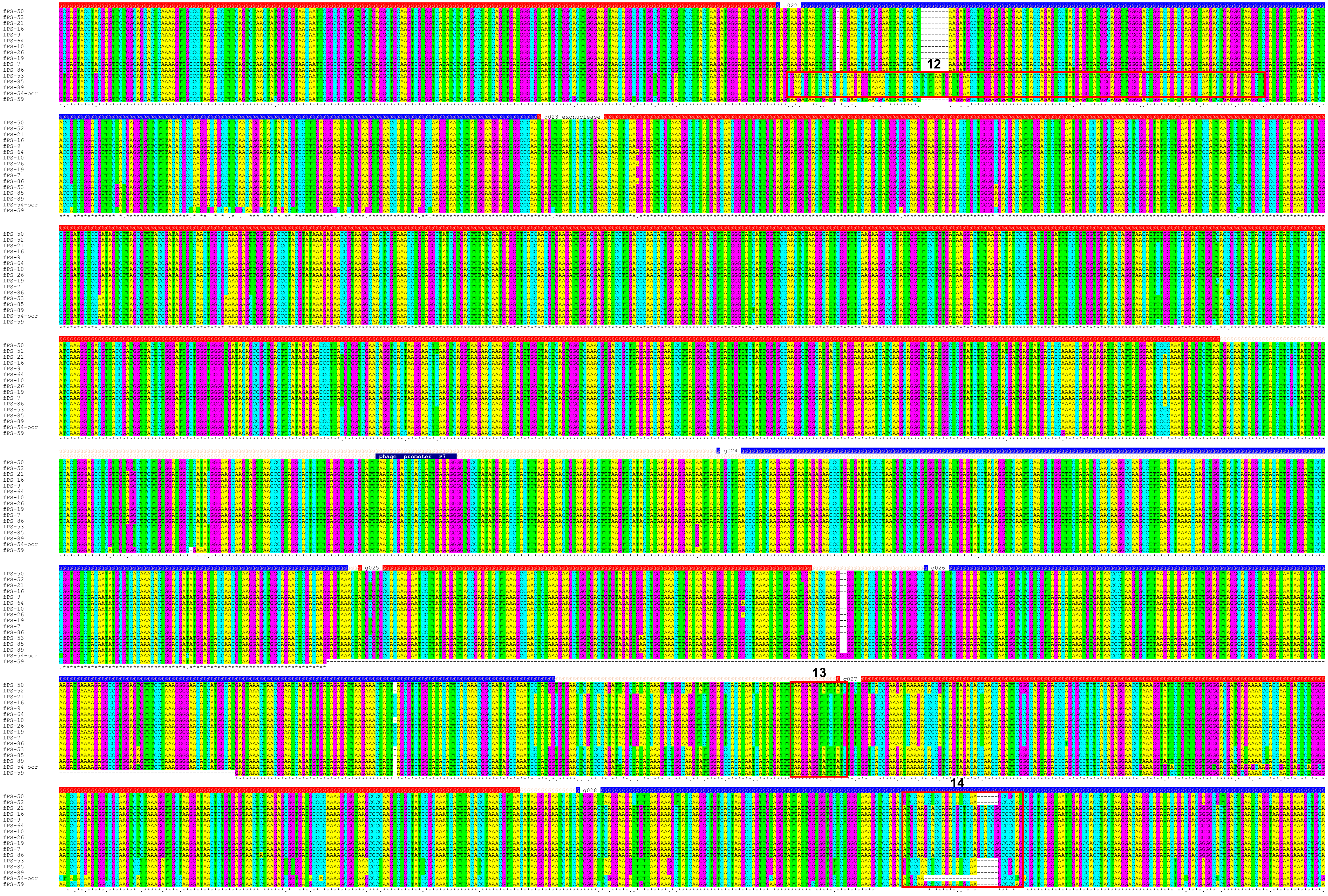
g022
12
g023 exonuclease
phage promoter P7
g024
g025
g026
13
g027
g028
14
fPS-50
fPS-52
fPS-21
fPS-16
fPS-9
fPS-64
fPS-10
fPS-26
fPS-19
fPS-7
fPS-86
fPS-53
fPS-85
fPS-89
fPS-54-ocr
fPS-59

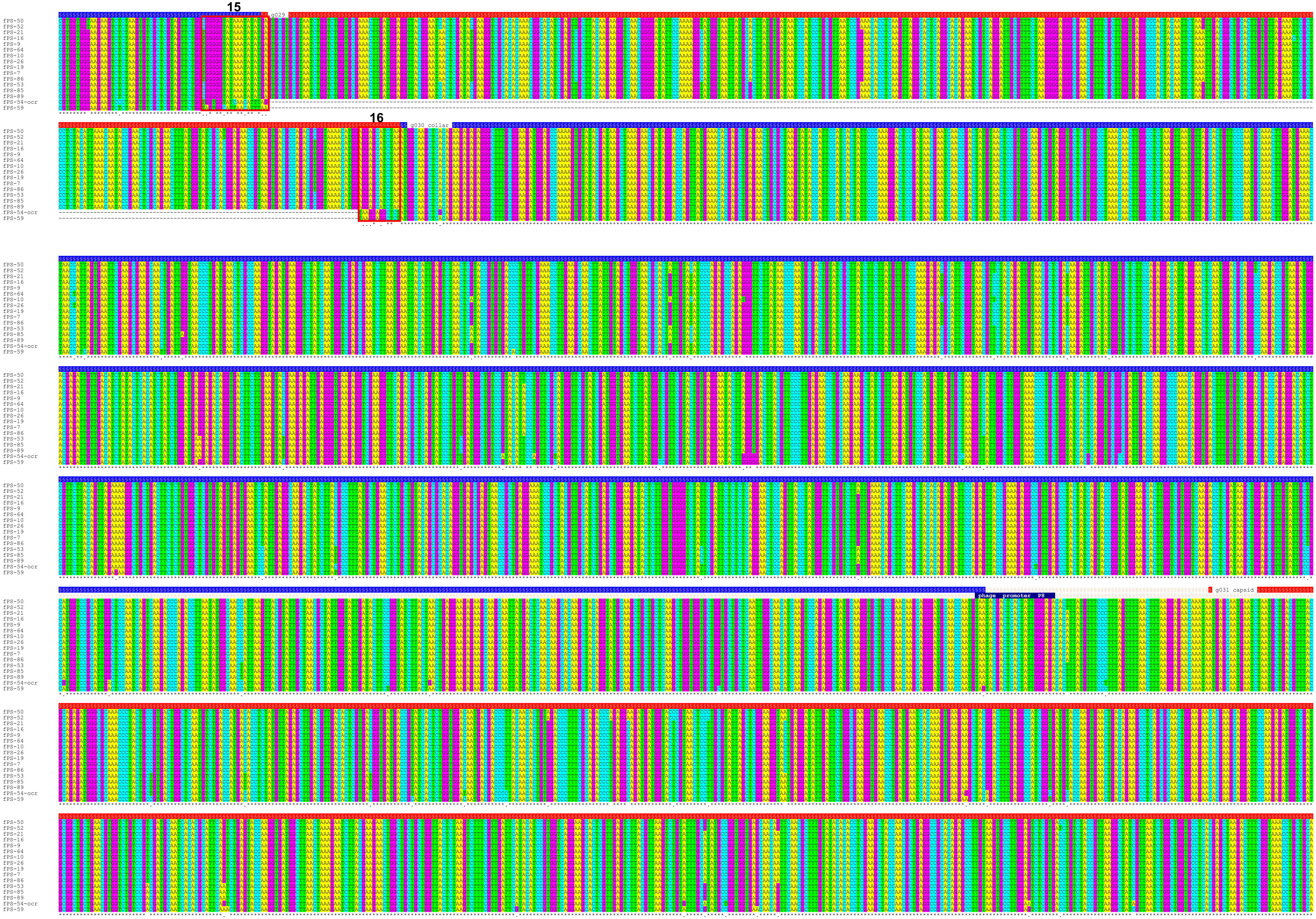
15
g029
16
g030 collar
phage promoter P8
g031 capsid
fPS-50
fPS-52
fPS-21
fPS-16
fPS-9
fPS-64
fPS-10
fPS-26
fPS-19
fPS-7
fPS-86
fPS-53
fPS-85
fPS-89
fPS-54-ocr
fPS-59

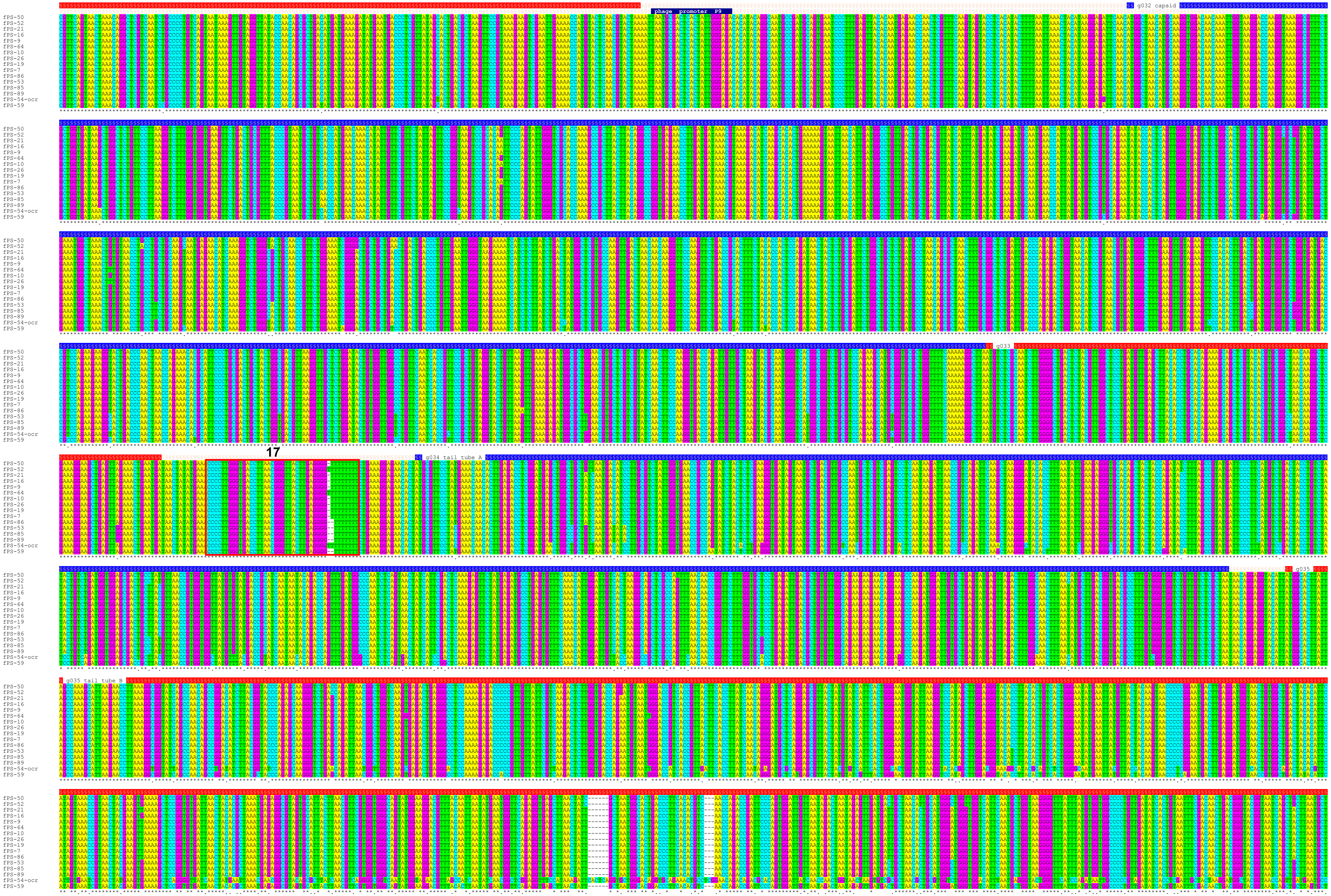
phage promoter P9
g032 capsid
g033
17
g034 tail tube A
g035
g035 tail tube B
fPS-50
fPS-52
fPS-21
fPS-16
fPS-9
fPS-64
fPS-10
fPS-26
fPS-19
fPS-7
fPS-86
fPS-53
fPS-85
fPS-89
fPS-54-ocr
fPS-59

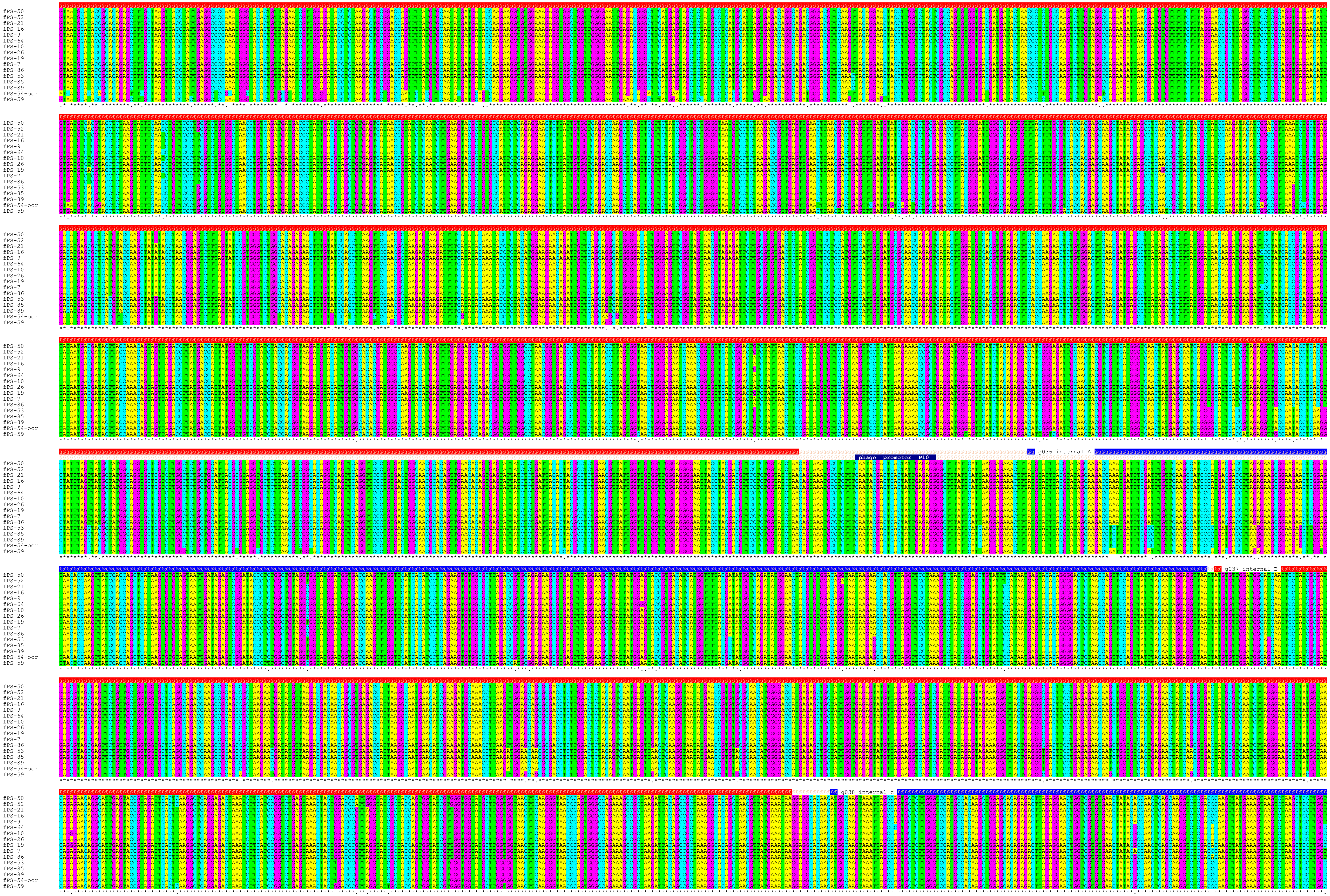
fPS-50
fPS-52
fPS-21
fPS-16
fPS-9
fPS-64
fPS-10
fPS-26
fPS-19
fPS-7
fPS-86
fPS-53
fPS-85
fPS-89
fPS-54-ocr
fPS-59
g036 internal A
phage promoter P10
g037 internal B
g038 internal c

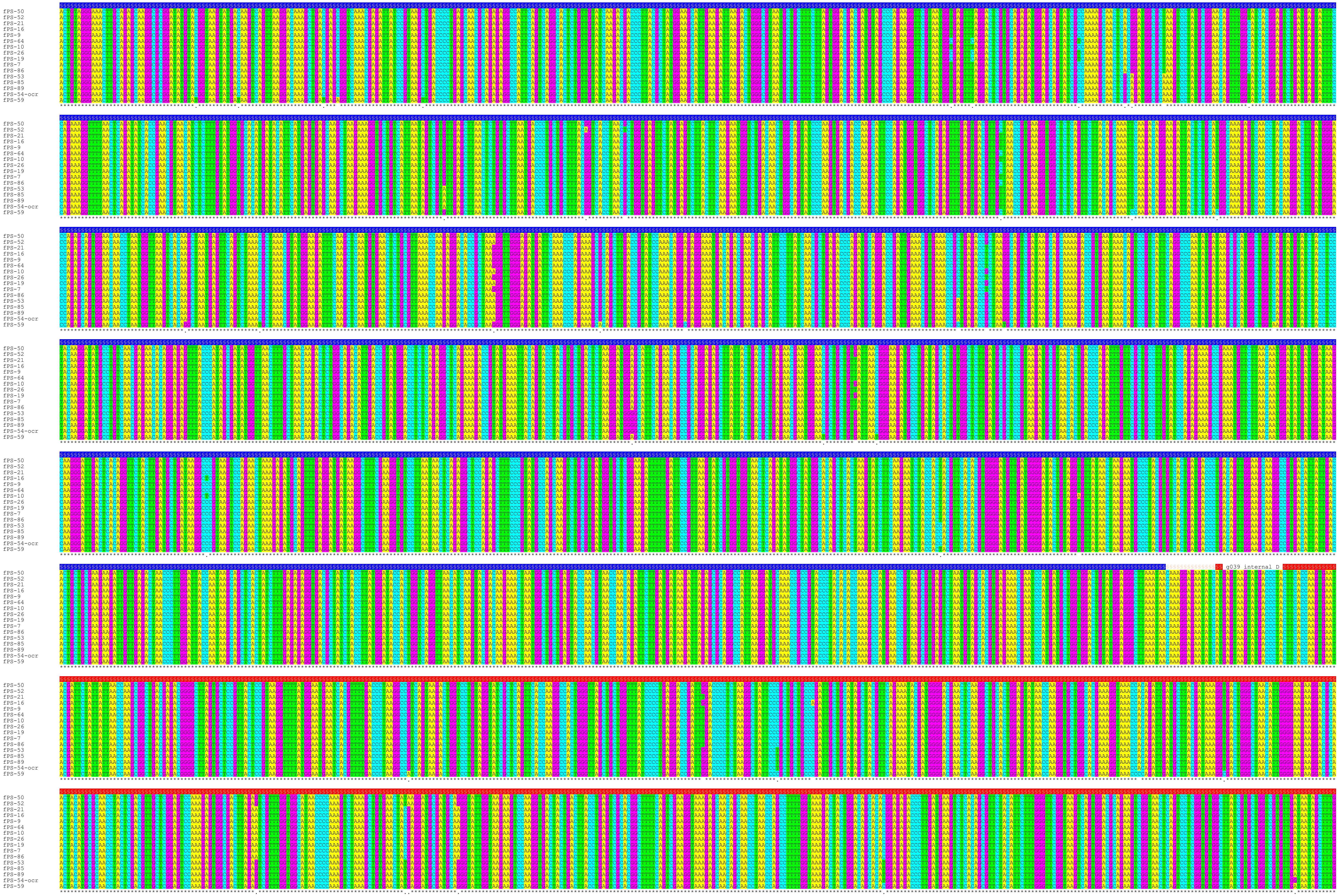
fPS-50
fPS-52
fPS-21
fPS-16
fPS-9
fPS-64
fPS-10
fPS-26
fPS-19
fPS-7
fPS-86
fPS-53
fPS-85
fPS-89
fPS-54-ocr
fPS-59
g039 internal D

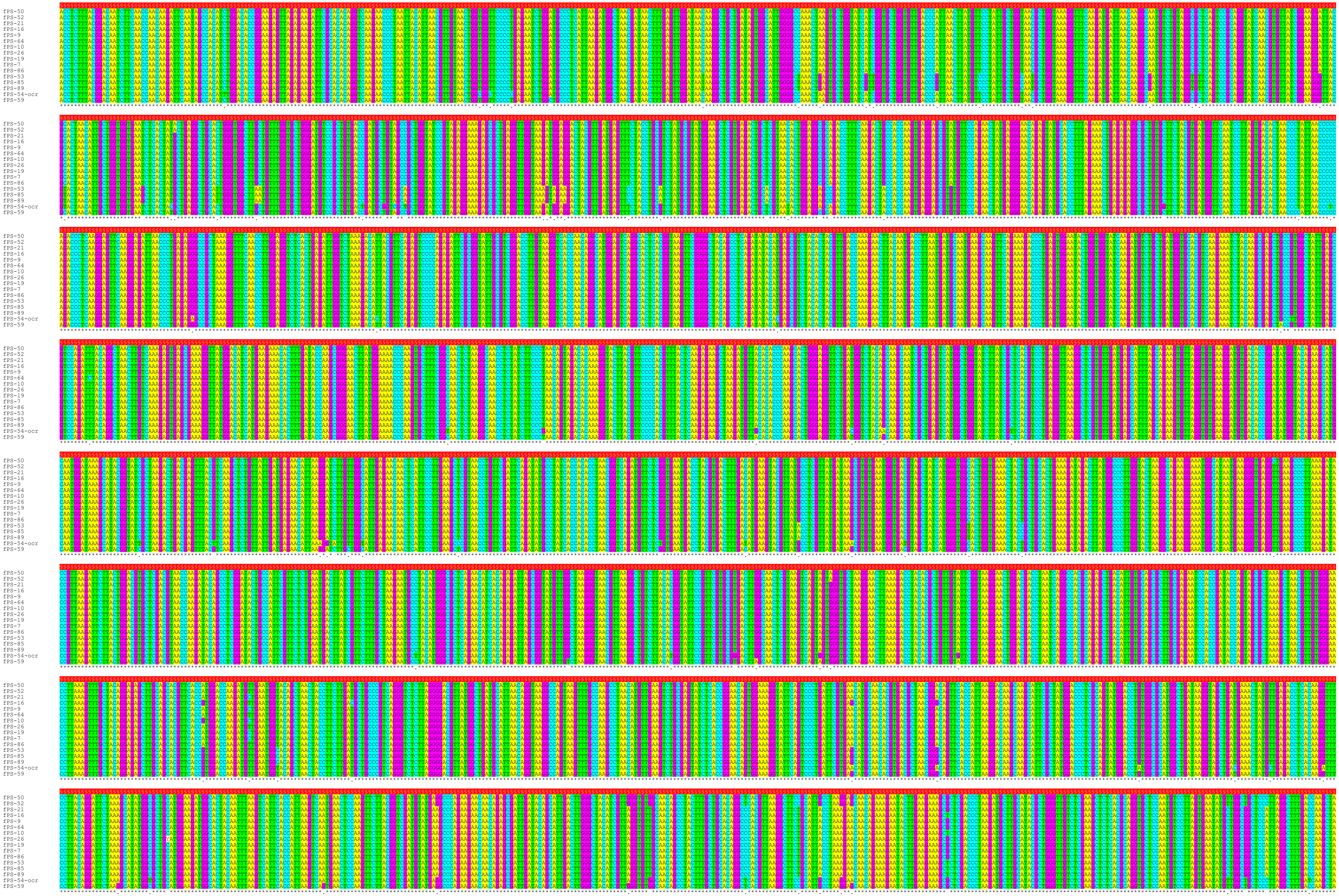
fPS-50
fPS-52
fPS-21
fPS-16
fPS-9
fPS-64
fPS-10
fPS-26
fPS-19
fPS-7
fPS-86
fPS-53
fPS-85
fPS-89
fPS-54-ocr
fPS-59

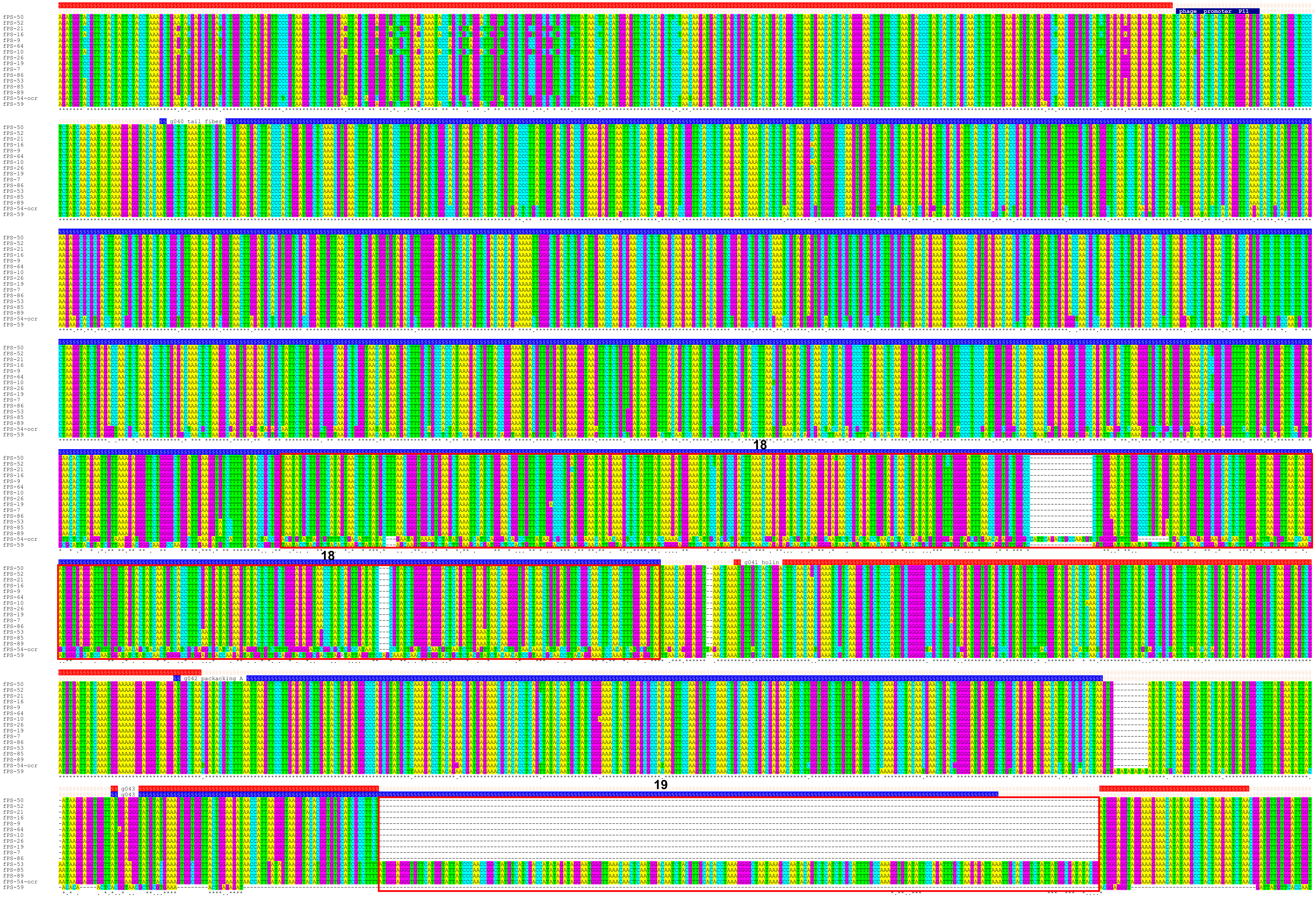
phage promoter P11
g040 tail fiber
g041 holin
g042 packacking A
g043
18
19
fPS-50
fPS-52
fPS-21
fPS-16
fPS-9
fPS-64
fPS-10
fPS-26
fPS-19
fPS-7
fPS-86
fPS-53
fPS-85
fPS-89
fPS-54-ocr
fPS-59

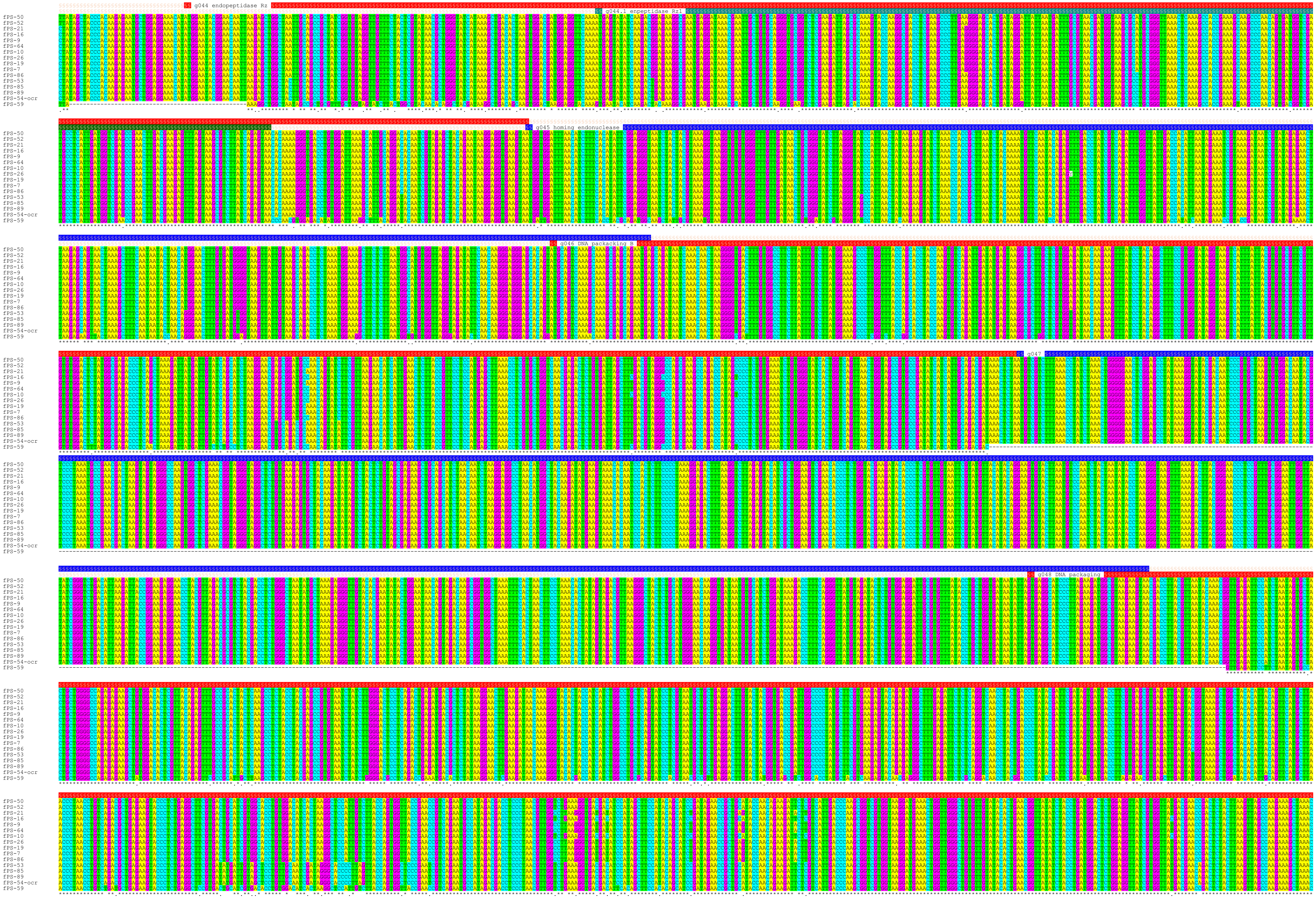

g044 endopeptidase Rz
g044.1 enpeptidase Rz1
g045 homing endonuclease
g046 DNA packacking B
g047
g048 DNA packaging
fPS-50
fPS-52
fPS-21
fPS-16
fPS-9
fPS-64
fPS-10
fPS-26
fPS-19
fPS-7
fPS-86
fPS-53
fPS-85
fPS-89
fPS-54-ocr
fPS-59

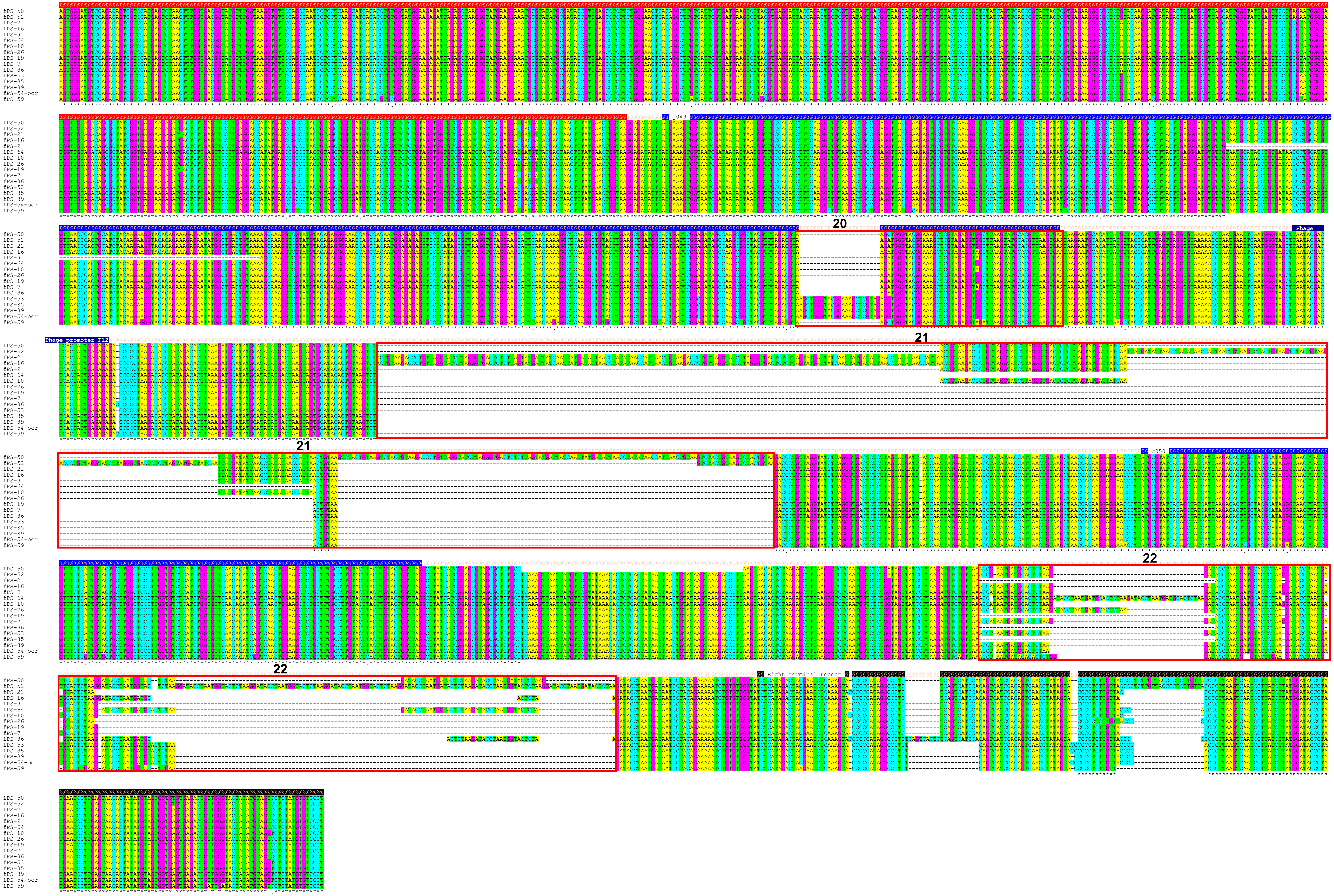

fPS-50
fPS-52
fPS-21
fPS-16
fPS-9
fPS-64
fPS-10
fPS-26
fPS-19
fPS-7
fPS-86
fPS-53
fPS-85
fPS-89
fPS-54-ocr
fPS-59
g049
20
Phage
21
Phage promoter P12
g050
22
Right terminal repeat

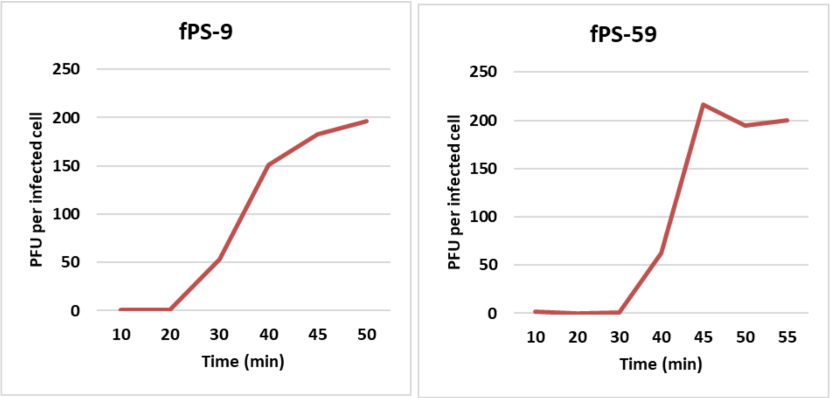

Figure S4. One-step growth curves of phages fPS-9 and fPS-59. Experiments were done in duplicate on at least two different occasions and the results of a representative experiment are shown.

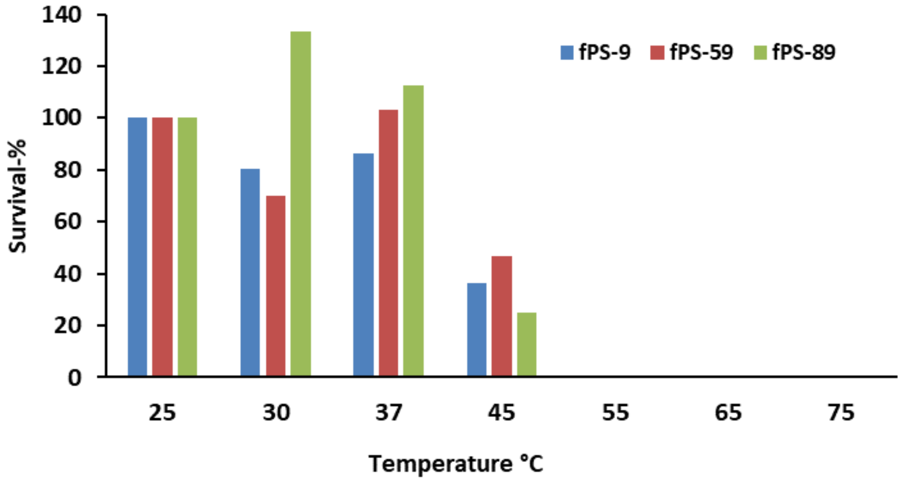

Figure S5. Thermal stability of phages fPS-9, fPS-59 and fPS-89. The PFU values of phages incubated at 25 °C were set to 100% and the survival-% of the phages at different temperatures were calculated relative to that. All values represent the means of two experiments.

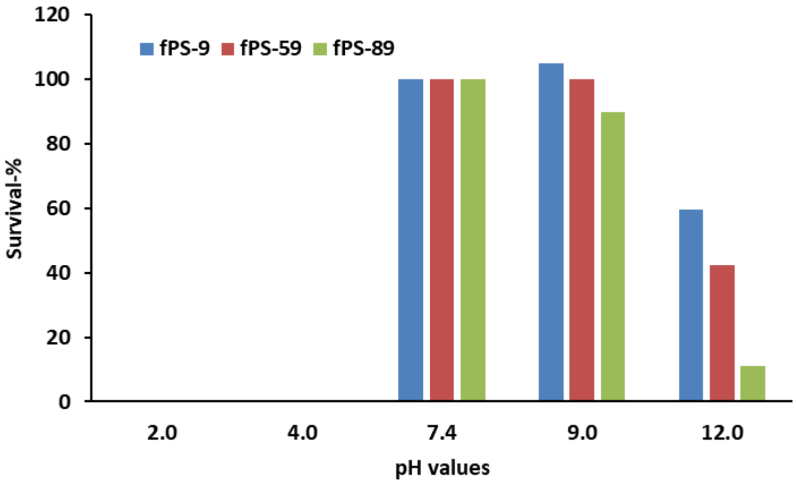

Figure S6. The effect of pH on the stability of phages fPS-9, fPS-59 and fPS-89. The PFU values of phages incubated at pH 7.4 were set to 100% and the survival-% of the phages exposed to different pH were calculated relative to that.

2. Supplementary tables

Table S1. Comparison of the amino acid sequences of the fPS-phage gene products to those of fPS-7. Also similarities to phage T7, T3 and ◎YeO3-12 gene products are indicated. Identity-% is indicated by different colors. Dark green = 100% identity; Green = 97-99% identity; lighter green = 94-96% identity; lightest green = 90-93% identity; light blue = 70-89 % identity; Light grey = 30-70% identity. Light gold = gene not present in fPS-7. HP = hypothetical protein.

Predicted function	Group Ib		Group Ia									Group II				Group III					
	fPS-50	fPS-52	fPS-64	fPS-10	fPS-9	fPS-16	fPS-21	fPS-7	fPS-19	fPS-86	fPS-26	fPS-53	fPS-89	fPS-85	fPS-54-ocr	fPS-59	Most closest hit in BLASTP	E-value	T7	T3	ϕYeO3-12
HP																Gp001	Nostoc piscinale HP, 54%	2e-22	-	-	-
HP	Gp001 (100%)	Gp001 (99%)	Gp001 (83%)	Gp001 (100%)	Gp001 (100%)	Gp001 (100%)	Gp001 (100%)	Gp001	Gp001 (100%)	Gp001 (100%)	Gp001 (100%)	Gp001 (83%)	Gp001 (83%)	Gp001 (83%)	Gp001 (83%)	Gp002 (85%)	Pectobacterium phage PPWS4, HP (36%)	1e-06	-	-	-
Protein kinase												Gp002 (100%)	Gp002 (100%)	Gp002 (100%)	Gp002 (100%)		Pectobacterium phage PP47, protein kinase (45%)	8e-65	Proteinkinase (45%)	Proteinkinase (44%)	Proteinkinase (43%)
RNA polymerase	Gp002 (99%)	Gp002 (99%)	Gp002 (99%)	Gp002 (100%)	Gp002 (100%)	Gp002 (99%)	Gp002 (99%)	Gp002	Gp002 (99%)	Gp002 (99%)	Gp002 (99%)	Gp003 (98%)	Gp003 (98%)	Gp003 (98%)	Gp003 (99%)	Gp003 (99%)	Erwinia phage FE44, RNA polymerase (83%)	0.0	Gp1, RNAP (73%)	RNAP (74%)	RNAP (74%)
HP	Gp003 (90%)	Gp003 (90%)	Gp003 (98%)	Gp003 (100%)	Gp003 (100%)	Gp003 (100%)	Gp003 (100%)	Gp003	Gp003 (98%)	Gp003 (98%)	Gp003 (98%)						Citrobacter phage SH1, HP (59%)	9e-07	-	-	-
HP	Gp004 (100%)	Gp004 (100%)															Citrobacter phage SH1, HP (60%)		-	-	-
HP	Gp005 (100%)	Gp005 (100%)	Gp004 (100%)	Gp004 (100%)	Gp004 (100%)	Gp004 (100%)	Gp004 (100%)	Gp004	Gp004 (100%)	Gp004 (100%)	Gp004 (100%)	Gp004 (100%)	Gp004 (100%)	Gp004 (100%)	Gp004 (100%)	Gp004 (100%)	Proteus phage vB_PmiP_Pm5460, HP (51%)	0.18	Gp 1.1 (46%)	-	-
HP	Gp006 (99%)	Gp006 (99%)	Gp005 (100%)	Gp005 (100%)	Gp005 (100%)	Gp005 (100%)	Gp005 (100%)	Gp005	Gp005 (99%)	Gp005 (100%)	Gp005 (100%)	Gp005 (94%)	Gp005 (95%)	Gp005 (94%)	Gp005 (94%)	Gp005 (95%)	No significant similarity	-	-	-	-
DNA ligase	Gp007 (100%)	Gp007 (99%)	Gp006 (99%)	Gp006 (100%)	Gp006 (100%)	Gp006 (100%)	Gp006 (100%)	Gp006	Gp006 (100%)	Gp006 (99%)	Gp006 (99%)	Gp006 (99%)	Gp006 (99%)	Gp006 (99%)	Gp006 (99%)	Gp006 (99%)	Yersinia phage vB_YenP_AP10, DNA ligase (58%)	9e-135	DNA ligase (57%)	-	DNA ligase (55%)
Phage protein	Gp008 (97%)	Gp008 (97%)	Gp007 (100%)	Gp007 (100%)	Gp007 (100%)	Gp007 (100%)	Gp007 (100%)	Gp007	Gp007 (100%)	Gp007 (98%)	Gp007 (100%)	Gp007 (95%)	Gp007 (95%)	Gp007 (95%)	Gp007 (95%)	Gp007 (97%)	Enterobacter phage E-3, HP (51%)	2e-22	HP (42%)	HP (48%)	-
HP	Gp009 (98%)	Gp009 (98%)	Gp008 (100%)	Gp008 (100%)	Gp008 (100%)	Gp008 (99%)	Gp008 (99%)	Gp008	Gp008 (99%)	Gp008 (100%)	Gp008 (100%)	Gp008 (99%)	Gp008 (99%)	Gp008 (99%)	Gp008 (99%)	Gp008 (98%)	Citrobacter phage CR8, HP (78%)	8e-45	-	-	-
Host RNA polymerase inhibitor	Gp010 (100%)	Gp010 (100%)	Gp009 (100%)	Gp009 (100%)	Gp009 (100%)	Gp009 (100%)	Gp009 (100%)	Gp009	Gp009 (100%)	Gp009 (100%)	Gp009 (100%)	Gp009 (100%)	Gp009 (100%)	Gp009 (100%)	Gp009 (100%)	Gp009 (100%)	Escherichia phage P483, host RNA polymerase inhibitor (68%)	2e-23	Host RNAP inh (61%)	Host RNAP inh (60%)	Host RNAP inh (52%)
Putative	Gp011 (99%)	Gp011 (99%)	Gp010 (99%)	Gp010 (100%)	Gp010 (100%)	Gp010 (100%)	Gp010 (100%)	Gp010	Gp010 (100%)	Gp010 (99%)	Gp010 (99%)	Gp010 (99%)	Gp010 (99%)	Gp010 (99%)	Gp010 (99%)	Gp010 (99%)	Pseudomonas sp. KGP01, HP (46%)	8e-26	-	-	-
ssDNA-binding protein	Gp012 (99%)	Gp012 (99%)	Gp011 (99%)	Gp011 (100%)	Gp011 (99%)	Gp011 (100%)	Gp011 (100%)	Gp011	Gp011 (99%)	Gp011 (99%)	Gp011 (99%)	Gp011 (98%)	Gp011 (98%)	Gp011 (98%)	Gp011 (98%)	Gp011 (97%)	Escherichia phage P694, ssDNA-binding protein (76%)	3e-105	Gp 2.5 (62%)	ssDNA-bp (58%)	ssDNA-bp (59%)
Phage protein																Gp012	Erwinia phage FE44, HP (74%)	2e-71	HP (74%)	-	-
Phage endonuclease	Gp013 (100%)	Gp013 (100%)	Gp012 (100%)	Gp012 (100%)	Gp012 (100%)	Gp012 (100%)	Gp012 (100%)	Gp012	Gp012 (100%)	Gp012 (100%)	Gp012 (100%)	Gp012 (100%)	Gp012 (100%)	Gp012 (100%)	Gp012 (99%)	Gp013 (76%)	Yersinia phage phiYeO3-12, endonuclease (88%)	1e-88	endonuclease (78%)	endonuclease (82%)	endonuclease (88%)
Phage lysin, N-AcMur-L-ala amidase	Gp014 (97%)	Gp014 (97%)	Gp013 (100%)	Gp013 (100%)	Gp013 (97%)	Gp013 (100%)	Gp013 (100%)	Gp013	Gp013 (97%)	Gp013 (100%)	Gp013 (99%)	Gp013 (100%)	Gp013 (100%)	Gp013 (100%)	Gp013 (100%)	Gp014 (93%)	Escherichia phage P694, N-AcMur-L-alanine amidase (86%)	2e-97	Gp 3.5 (70%)	-	N-AcMur-L-ala amidase (72%)
Homing endonuclease	Gp015 (99%)	Gp015 (98%)	Gp014 (100%)	Gp014 (100%)	Gp014 (100%)	Gp014 (100%)	Gp014 (100%)	Gp014	Gp014 (100%)	Gp014 (100%)	Gp014 (100%)	Gp014 (96%)	Gp014 (96%)	Gp013.1 (96%)	Gp014 (96%)	Gp015 (93%)	Enterobacteria phage T7, HP (74%)	5e-61	Gp 3.8 (73%)	-	-
Primase / helicase	Gp016 (99%)	Gp016 (99%)	Gp015 (100%)	Gp015 (99%)	Gp015 (100%)	Gp015 (100%)	Gp015 (100%)	Gp015	Gp015 (100%)	Gp015 (100%)	Gp015 (100%)	Gp015 (99%)	Gp015 (99%)	Gp014 (99%)	Gp015 (99%)	Gp016 (99%)	Yersinia phage Berlin, Helicase (77%)	0.0	DNA primase / helicase (69%)	helicase (72%)	DNA primase / helicase (70%)
HP	Gp017 (100%)	Gp017 (100%)	Gp016 (100%)	Gp016 (100%)	Gp016 (100%)	Gp016 (100%)	Gp016 (100%)	Gp016	Gp016 (100%)	Gp016 (100%)	Gp016 (100%)	Gp016 (100%)	Gp016 (100%)	Gp015 (100%)	Gp015.1 100%	Gp017 (95%)	No significant similarity		-	-	-
HP	Gp018 (100%)	Gp018 (100%)	Gp017 (100%)	Gp017 (100%)	Gp017 (100%)	Gp017 (100%)	Gp017 (100%)	Gp017	Gp017 (100%)	Gp017 (100%)	Gp017 (100%)	Gp017 (100%)	Gp017 (100%)	Gp016 (100%)	Gp016 (100%)	Gp018 (99%)	Morganella phage vB_MmoP_MP2, HP (45%)	3e-07	HP (43%)	-	-
Phage protein	Gp019 (100%)	Gp019 (100%)	Gp018 (100%)	Gp018 (100%)	Gp018 (100%)	Gp018 (100%)	Gp018 (100%)	Gp018	Gp018 (100%)	Gp018 (100%)	Gp018 (100%)	Gp018 (98%)	Gp018 (98%)	Gp017 (98%)	Gp017 (100%)	Gp019 (100%)	Escherichia phage P694, HP (76%)	3e-38	HP (63%)	gene 4.5 (54%)	-
Phage protein												Gp019 (100%)	Gp019 (100%)	Gp018 (100%)	Gp018 (100%)	Gp020 (95%)	Kluyvera phage Kvp1, phage protein (42%)	2e-18	HP (39%)	-	-
Phage DNA polymerase	Gp020 (99%)	Gp020 (99%)	Gp019 (99%)	Gp019 (99%)	Gp019 (100%)	Gp019 (100%)	Gp019 (99%)	Gp019	Gp019 (99%)	Gp019 (99%)	Gp019 (99%)	Gp020 (98%)	Gp020 (98%)	Gp019 (98%)	Gp019 (98%)	Gp021 (98%)	Kluyvera phage Kvp1, DNA polymerase (83%)	0.0	DNA pol (71%)	DNA pol (71%)	DNA pol (69%)
HNS binding protein	Gp021 (100%)	Gp021 (100%)	Gp020 (100%)	Gp020 (100%)	Gp020 (100%)	Gp020 (100%)	Gp020 (100%)	Gp020	Gp020 (100%)	Gp020 (99%)	Gp020 (100%)	Gp021 (81%)	Gp021 (81%)	Gp020 (81%)	Gp020 (81%)	Gp022 (84%)	Leclercia phage 10164-302, HP (38%)	3e-08	-	gene 5.5 (37%)	HP (37%)
HNS binding protein	Gp022 (100%)	Gp022 (100%)	Gp021 (100%)	Gp021 (100%)	Gp021 (100%)	Gp021 (100%)	Gp021 (100%)	Gp021	Gp021 (100%)	Gp021 (100%)	Gp021 (100%)	Gp022 (100%)	Gp022 (100%)	Gp021 (100%)	Gp021 (100%)	Gp023 (99%)	Salmonella phage BP12A, HP (91%)	2e-40	Gp 5.7 (75%)	-	Gp 5.7 (81%)
HP	Gp023 (100%)	Gp023 (100%)	Gp022 (100%)	Gp022 (100%)	Gp022 (100%)	Gp022 (100%)	Gp022 (100%)	Gp022	Gp022 (100%)	Gp022 (100%)	Gp022 (100%)	Gp023 (82%)	Gp023 (82%)	Gp022 (82%)	Gp022 (82%)	Gp024 (82%)	Salmonella phage BP12A, HP (71%)	5e-35	-	-	-
Phage exonuclease	Gp024 (100%)	Gp024 (100%)	Gp023 (100%)	Gp023 (100%)	Gp023 (100%)	Gp023 (100%)	Gp023 (100%)	Gp023	Gp023 (100%)	Gp023 (100%)	Gp023 (100%)	Gp024 (100%)	Gp024 (100%)	Gp023 (100%)	Gp023 (100%)	Gp025 (99%)	Escherichia phage P694, exonuclease (76%)	3e-167	exonuclease (69%)	exonuclease (68%)	exonuclease (73%)
HP																Gp026	No similarity	-	-	-	-
Phage protein	Gp025 (100%)	Gp025 (100%)	Gp025 (100%)	Gp024 (100%)	Gp024 (100%)	Gp024 (100%)	Gp024 (100%)	Gp024	Gp024 (100%)	Gp024 (100%)	Gp024 (100%)	Gp025 (100%)	Gp025 (100%)	Gp024 (100%)	Gp024 (100%)	Gp027 (100%)	Salmonella phage BP12A, HP (73%)	2e-34	HP (45%)	HP (48%)	Gp 6.5 (50%)
HP (?homing endonuclease)	Gp026 (100%)	Gp026 (100%)															Enterobacteria phage 13a, gp7.7 (50%)	1e-34	HP (45%)	-	-
HP			Gp026 (95%)	Gp025 (98%)	Gp025 (100%)	Gp025 (100%)	Gp025 (100%)	Gp025	Gp025 (100%)	Gp025 (100%)	Gp025 (95%)						Branchiostoma floridae, HP (44%)	0.056	-	-	-
Phage protein			Gp027 (100%)	Gp026 (97%)	Gp026 (100%)	Gp026 (97%)	Gp026 (100%)	Gp026	Gp026 (100%)	Gp026 (100%)	Gp026 (100%)	Gp026 (96%)	Gp026 (96%)	Gp025 (96%)	Gp025 (97%)		Pectobacterium phage PP74, HNH homing endonuclease (65%)	6e-16	HP (53%)	-	-
Phage protein			Gp028 (100%)	Gp027 (100%)	Gp027 (100%)	Gp027 (100%)	Gp027 (100%)	Gp027	Gp027 (100%)	Gp027 (100%)	Gp027 (100%)						Erwinia phage FE44, HP (66%)	2e-19	HP (55%)	HP (51%)	HP (51%)
Phage protein	Gp027 (87%)	Gp027 (88%)	Gp029 (100%)	Gp028 (100%)	Gp028 (100%)	Gp028 (100%)	Gp028 (100%)	Gp028	Gp028 (100%)	Gp028 (100%)	Gp028 (100%)	Gp027 (86%)	Gp027 (86%)	Gp026 (86%)	Gp026 (88%)	Gp028 (91%)	Yersinia phage Yep-phi, HP (62%)	1e-24	host range (51%)	host range (71%)	host range (49%)
Phage protein	Gp028 (98%)	Gp028 (98%)	Gp030 (100%)	Gp029 (100%)	Gp029 (100%)	Gp029 (99%)	Gp029 (100%)	Gp029	Gp029 (100%)	Gp029 (100%)	Gp029 (100%)	Gp028 (98%)	Gp028 (98%)	Gp027 (98%)			Escherichia phage vB_EcoP_GA2A, HP (50%)	2e-39	HP (44%)	-	-
Head-to-tail joining protein	Gp029 (100%)	Gp029 (100%)	Gp031 (100%)	Gp030 (99%)	Gp030 (100%)	Gp030 (100%)	Gp030 (100%)	Gp030	Gp030 (100%)	Gp030 (100%)	Gp030 (100%)	Gp029 (100%)	Gp029 (100%)	Gp028 (99%)	Gp027 (99%)	Gp029 (99%)	Salmonella phage BP12A, head to tail joining protein (88%)	0.0	head to tail (80%)	head to tail (83%)	head to tail (83%)
Phage capsid and scaffolding protein	Gp030 (99%)	Gp030 (99%)	Gp032 (100%)	Gp031 (100%)	Gp031 (100%)	Gp031 (100%)	Gp031 (100%)	Gp031	Gp031 (100%)	Gp031 (100%)	Gp031 (100%)	Gp030 (99%)	Gp030 (99%)	Gp029 (99%)	Gp028 (99%)	Gp030 (99%)	Escherichia phage P694, capsid and scaffold protein (64%)	1e-127	Gp 9 (51%)	Gp 9 (54%)	capsid protein (53%)
Phage capsid and scaffolding protein	Gp031 (99%)	Gp031 (99%)	Gp033 (100%)	Gp032 (100%)	Gp032 (100%)	Gp032 (100%)	Gp032 (100%)	Gp032	Gp032 (100%)	Gp032 (100%)	Gp032 (100%)	Gp031 (99%)	Gp031 (99%)	Gp030 (99%)	Gp029 (99%)	Gp031 (99%)	Escherichia phage P694, , capsid and scaffold protein (86%)	0.0	minor capsid 10B (76%)	minor capsid 10B (78%)	minor capsid 10B (78%)
HP	Gp032 (100%)	Gp032 (100%)	Gp034 (100%)	Gp033 (100%)	Gp033 (100%)	Gp033 (100%)	Gp033 (100%)	Gp033	Gp033 (100%)	Gp033 (100%)	Gp033 (100%)	Gp032 (100%)		Gp031 (100%)	Gp030 (100%)		Enterobacteria phage 285P, gp10b (69%)	2e-05	predicted protein (58%)	-	-
Tail tubular protein A	Gp033 (98%)	Gp033 (99%)	Gp035 (100%)	Gp034 (100%)	Gp034 (100%)	Gp034 (100%)	Gp034 (100%)	Gp034	Gp034 (100%)	Gp034 (100%)	Gp034 (100%)	Gp033 (99%)	Gp033 (99%)	Gp032 (99%)	Gp031 (98%)	Gp033 (99%)	Escherichia phage P483, tail tubular protein A (86%)	1e-124	Gp 11 (68%)	tail tubular proteinA (73%)	-
Tail tubular protein B	Gp034 (99%)	Gp034 (99%)	Gp036 (99%)	Gp035 (100%)	Gp035 (100%)	Gp035 (100%)	Gp035 (100%)	Gp035	Gp035 (100%)	Gp035 (100%)	Gp035 (100%)	Gp034 (89%)	Gp034 (99%)	Gp033 (99%)	Gp032 (92%)	Gp034 (98%)	Erwinia phage FE44, tail tubular protein B (74%)	0.0	tail tubular protein B (62%)	tail tubular protein B (60%)	tail tubular protein B (61%)
Internal (core) protein A	Gp035 (100%)	Gp035 (99%)	Gp037 (99%)	Gp036 (100%)	Gp036 (100%)	Gp036 (100%)	Gp036 (100%)	Gp036	Gp036 (100%)	Gp036 (100%)	Gp036 (100%)	Gp035 (96%)	Gp035 (96%)	Gp034 (96%)	Gp033 (96%)	Gp035 (99%)	Yersinia phage Yepe2, gp13 (69%)	4e-62	Gp 13 (57%)	-	internal virion proteinA (53%)
Internal (core) protein B	Gp036 (100%)	Gp036 (100%)	Gp038 (100%)	Gp037 (100%)	Gp037 (100%)	Gp037 (100%)	Gp037 (100%)	Gp037	Gp037 (100%)	Gp037 (100%)	Gp037 (100%)	Gp036 (98%)	Gp036 (98%)	Gp035 (98%)	Gp034 (98%)	Gp036 (99%)	Kluyvera phage Kvp1, internal virion protein B (58%)	4e-70	internal virion protein B (44%)	-	internal virion protein B (48%)
Internal (core) protein C	Gp037 (99%)	Gp037 (99%)	Gp039 (99%)	Gp038 (99%)	Gp038 (100%)	Gp038 (100%)	Gp038 (100%)	Gp038	Gp038 (100%)	Gp038 (99%)	Gp038 (99%)	Gp037 (99%)	Gp037 (99%)	Gp036 (99%)	Gp035 (99%)	Gp037 (99%)	Pectobacterium phage PP74, internal virion protein C (74%)	0.0	internal virion protein C (59%)	protein inside capsid C (60%)	internal virion protein C (60%)
Internal (core) protein D	Gp038 (99%)	Gp038 (99%)	Gp040 (100%)	Gp039 (99%)	Gp039 (100%)	Gp039 (99%)	Gp039 (100%)	Gp039	Gp039 (100%)	Gp039 (100%)	Gp039 (99%)	Gp038 (99%)	Gp038 (99%)	Gp037 (99%)	Gp036 (99%)	Gp038 (99%)	Salmonella phage BP12A, DNA injection channel protein C (70%)	0.0	internal virion protein D (65%)	internal virion prot D (64%)	internal virion prot D (65%)
Phage tail fibers	Gp039 (99%)	Gp039 (99%)	Gp041 (100%)	Gp040 (99%)	Gp040 (100%)	Gp040 (99%)	Gp040 (99%)	Gp040	Gp040 (99%)	Gp040 (100%)	Gp040 (100%)	Gp039 (99%)	Gp039 (99%)	Gp038 (99%)	Gp037 (74%)	Gp039 (75%)	Escherichia phage ECA2, tail fiber protein (58%)	2e-110	tail fiber protein (51%)	tail fiber protein (52%)	tail fiber protein (61%)
Phage holin	Gp040 (99%)	Gp040 (99%)	Gp042 (100%)	Gp041 (100%)	Gp041 (100%)	Gp041 (100%)	Gp041 (100%)	Gp041	Gp041 (100%)	Gp041 (100%)	Gp041 (100%)	Gp040 (100%)	Gp040 (100%)	Gp039 (100%)	Gp038 (91%)	Gp040 (100%)	Salmonella phage BP12A, lysin protein (87%)	2e-34	lysis protein (72%)	lysis protein (72%)	lysis protein (70%)
DNA packaging protein A	Gp041 (100%)	Gp041 (100%)	Gp043 (100%)	Gp042 (100%)	Gp042 (100%)	Gp042 (100%)	Gp042 (100%)	Gp042	Gp042 (100%)	Gp042 (100%)	Gp042 (100%)	Gp041 (100%)	Gp041 (100%)	Gp040 (100%)	Gp039 (99%)	Gp041 (99%)	Yersinia phage Berlin, DNA packaging protein A (83%)	1e-44	protein 18 (66%)	DNA pack A (69%)	DNA pack A (73%)
HP	Gp042 (100%)	Gp042 (100%)	Gp043.1 (92%)	Gp043 (100%)	Gp043 (100%)	Gp043 (100%)	Gp043 (100%)	Gp043	Gp043 (100%)	Gp043 (100%)	Gp043 (100%)	Gp042 (93%)	Gp042 (93%)	Gp041 (93%)	Gp040 (93%)		Pseudomonas sp. Lz4W, HNH endonuclease (44%)	2.1			
Endopeptidase Rz	Gp043 (100%)	Gp043 (100%)	Gp044 (100%)	Gp044 (100%)	Gp044 (100%)	Gp044 (100%)	Gp044 (100%)	Gp044	Gp044 (100%)	Gp044 (99%)	Gp044 (100%)	Gp043 (99%)	Gp043 (99%)	Gp042 (98%)	Gp041 (99%)	Gp042 (83%)	Enterobacteria phage 285P (62%) gp18.5	4e-56	endopeptidase (52%)	endopeptidase (51%)	endopeptidase (40%)
Endopeptidase Rz1	Gp043.1 (99%)	Gp043.1 (100%)	Gp044.1 (100%)	Gp044.1 (100%)	Gp044.1 (100%)	Gp044.1 (100%)	Gp044.1 (100%)	Gp044.1	Gp044.1 (100%)	Gp044.1 (99%)	Gp044.1 (100%)	Gp043.1 (98%)	Gp043.1 (98%)	Gp042.1 (94%)	Gp042 (98%)	Gp042.1 (92%)	Enterobacteria phage BA14, gp18.7 (62%)	1e-17	gp18.7 (49%)	gene 18.7 protein (44%)	18.7 protein (51%)
Homing endonuclease	Gp044 (100%)	Gp044 (100%)	Gp045 (100%)	Gp045 (100%)	Gp045 (100%)	Gp045 (100%)	Gp045 (100%)	Gp045	Gp045 (100%)	Gp045 (100%)	Gp045 (99%)	Gp044 (98%)	Gp044 (98%)	Gp043 (98%)	Gp043 (98%)	Gp043 (90%)	Salmonella phage LPST10, homing endonuclease (38%)	5e-15			
DNA packaging protein B	Gp045 (100%)	Gp045 (100%)	Gp046 (100%)	Gp046 (100%)	Gp046 (100%)	Gp046 (100%)	Gp046 (100%)	Gp046	Gp046 (100%)	Gp046 (100%)	Gp046 (100%)	Gp045 (100%)	Gp045 (100%)	Gp044 (100%)	Gp044 (100%)	Gp044 (100%)	Yersinia phage vB_YenP_AP10, DNA packaging protein B (89%)	3e-97	DNA packaging protein B (87%)	DNA packaging protein B (88%)	DNA packaging protein B (87%)
HP	Gp046 (100%)	Gp046 (100%)	Gp047 (100%)	Gp047 (100%)	Gp047 (100%)	Gp047 (100%)	Gp047 (100%)	Gp047	Gp047 (100%)	Gp047 (100%)	Gp047 (100%)	Gp046 (100%)	Gp046 (100%)	Gp045 (100%)	Gp045 (100%)		Salmonella phage Vi06, conserved HP (73%)	4e-90	-	-	-
Phage DNA packaging	Gp047 (99%)	Gp047 (99%)	Gp048 (99%)	Gp048 (99%)	Gp048 (100%)	Gp048 (99%)	Gp048 (99%)	Gp048	Gp048 (99%)	Gp048 (99%)	Gp048 (99%)	Gp047 (99%)	Gp047 (99%)	Gp046 (99%)	Gp046 (99%)		Escherichia phage P483, DNA packaging protein (80%)	0.0	DNA packaging protein B (75%)	DNA packaging protein B (74%)	DNA packaging protein B (75%)
Phage protein	Gp048 (98%)	Gp048 (98%)	Gp049 (98%)	Gp049 (98%)	Gp049 (100%)	Gp049 (98%)	Gp049 (98%)	Gp049	Gp049 (98%)	Gp049 (98%)	Gp049 (98%)	Gp048 (98%)	Gp048 (98%)	Gp047 (98%)	Gp047 (98%)	Gp045 (97%)	Salmonella phage BP12A, HP (68%)	1e-73			
Phage protein					Gp050												Yersinia phage Berlin, phage protein (68%)	3e-16	-	-	-
Phage protein	Gp049 (100%)	Gp049 (100%)	Gp050 (100%)	Gp050 (100%)	Gp051 (100%)	Gp050 (100%)	Gp050 (100%)	Gp050	Gp050 (100%)	Gp050 (100%)	Gp050 (100%)	Gp049 (100%)	Gp049 (100%)	Gp048 (100%)	Gp048 (100%)	Gp046 (96%)	Yersinia phage YpP-G, HP (59%)	7e-14	HP (51%)	gp 19.5 (46%)	gp 19.5 (49%)

Table S2. Whole genome DNA sequence comparisons of fPS-phages using the EMBOSS stretcher program. Phages falling into same species (sharing >95% identity between them) are similarly colored. The grey cells indicate identities <95%.

	fPS-7	fPS-9	fPS-10	fPS-16	fPS-19	fPS-21	fPS-26	fPS-50	fPS-52	fPS-64	fPS-86	fPS-53	fPS-54-ocr	fPS-85	fPS-89	fPS-59
**fPS-7**	100.0.0															
**fPS-9**	99.3	100.0														
**fPS-10**	99.2	99.3	100.0													
**fPS-16**	99.1	98.8	99.1	100.0												
**fPS-19**	99.7	99.2	99.0	98.9	100.0											
**fPS-21**	99.2	98.9	98.9	99.7	99.2	100.0										
**fPS-26**	99.5	99.1	99.2	98.8	99.4	98.8	100.0									
**fPS-50**	96.8	96.9	97.2	97.3	96.9	97.1	97.0	100.0								
**fPS-52**	96.9	97.0	97.1	97.3	96.9	97.1	97.0	99.2	100.0							
**fPS-64**	98.9	98.5	98.8	98.2	98.8	98.2	99.3	96.9	96.8	100.0						
**fPS-86**	99.6	99.0	99.0	98.9	99.5	99.0	99.6	96.9	96.9	99.0	100.0					
**fPS-53**	92.9	92.6	92.8	92.4	92.9	92.4	93.1	91.2	91.4	92.5	93.1	100.0				
**fPS-54-ocr**	90.3	89.9	90.2	89.9	90.2	89.7	90.4	88.7	88.8	89.9	90.4	96.8	100.0			
**fPS-85**	93.0	92.6	92.8	92.4	92.9	92.4	93.0	91.2	91.3	92.5	93.0	99.9	96.7	100.0		
**fPS-89**	93.0	92.6	92.7	92.5	93,0	92.5	93.0	91.3	91.3	92.4	93.1	99.9	96.7	99.8	100.0	
**fPS-59**	87.4	87.2	87.5	87.0	87.4	86.9	87.6	86.0	86.1	87.2	87.5	88.5	88.9	88.4	88.4	100.0

Table S3. Predicted promoter and terminator sequences and locations of Yersinia phage fPS-7

**Host RNAP promoters (-35 and -10 boxes)**	**5′-end pos.**	**Downstream gene (product)**
TCCACA $(N)_{14}$ ACCTATAGT	49	*g001* (hypothetical protein)
TTAAAG $(N)_{14}$ TCTTAAAGT	549	*g001* (hypothetical protein)
ATGGAA $(N)_{12}$ GCTTATTAT	867	*g002* (RNA polymerase),within *g001*
TTGCAG $(N)_{18}$ GGTTATATT	1179	*g002* (RNA polymerase)
**Phage RNAP promoters**		
TAATACAACTCACTATTGAGAGA	471	*g001* (hypothetical protein)
TAATAGAACTCACTATTGGGAGC	3963	*g003* (hypothetical protein)
TAATACAACTCACTATTGAGAAA	4237	*g004* (hypothetical protein)
TAATGGAACTCACTATTGGGAGA	5924	*g007* (hypothetical protein)
TAATAGAACTCACTATTGAGAAC	7348	*g011* (ssDNA-binding protein)
TAATAGAACTCACTATTGGGAGA	9014	*g014* (homing endonuclease)
TAATACGACTCACTATTGAGAGG	15834	*g024* (phage protein)
TAATACGACTCACTATTGGGAGA	19245	*g031* (capsid protein)
TAATGCGACTCACTATTGGGAGA	20230	*g032* (capsid protein)
CAATACGACTCACTATTGAGAGG	24598	*g036* (core protein)
CAATACGACTCACTATTGGGAGT	31893	*g040* (tail fiber protein)
TAATACGACTCACTATTGAGAGA	38139	*g050* (phage protein)
**Consensus promoter**		
TAATACGACTCACTATTGGGAGA		
**Rho-independent terminators (hairpin stem in blue and loop in red)**		
AGTACACACATGGTCAACCTACGGGTTGGCCTTTTTGCGTTTT	5882	*g007*
GACTCACTATTGGGAGACACATTATGTTTCCCTTTAGTTTTAAC	19251	*g031*
AACTATATGAAACCCCTTGGGTGACCTTAACGGGTTACTTGAGGGGTTTTTTTTCTGAAA	21532	*g034*
TAACAGTAAATGCCTCTTTCAATACGACTCACTATTGAGAGGGGCtTTATTCATTAAGG	24579	*g036*

Table S4. Predicted promoter and terminator sequences and locations of Yersinia phage fPS-9

**Host RNAP promoters (-35 and -10 boxes)**	**5′-end pos.**	**Downstream gene (product)**
TCCACA $(N)_{14}$ ACCTATAGT	49	*g001* (hypothetical protein)
TTAAAG $(N)_{14}$ TCTTAAAGT	626	*g001* (hypothetical protein)
GTGATT $(N)_{15}$ CAGTATACT	1046	*g002* (RNA polymerase)
**Phage RNAP promoters**		
TAATACAACTCACTATTGAGAGA	548	*g001* (hypothetical protein)
TAATACAACTCACTATTGAGAGA	847	*g001* (hypothetical protein)
TAATAGAACTCACTATTGGGAGC	4040	*g003* (hypothetical protein)
TAATACAACTCACTATTGAGAAA	4314	*g004* (hypothetical protein)
TAATGGAACTCACTATTGGGAGA	6001	*g007* (phage protein)
TAATAGAACTCACTATTGAGAAC	7425	*g011* (ssDNA-binding protein)
TAATAGAACTCACTATTGGGAGA	9091	*g014* (homing endonuclease)
TAATACGACTCACTATTGAGAGG	15908	*g024* (phage protein)
TAATACGACTCACTATTGGGAGA	19319	*g031* (capsid protein)
TAATGCGACTCACTATTGGGAGA	20304	*g032* (capsid protein)
CAATACGACTCACTATTGAGAGG	24672	*g036* (core protein)
CAATACGACTCACTATTGGGAGT	31967	*g040* (tail fiber protein)
TAATACGACTCACTATTGAGAGA	38126	*g051* (phage protein)
**Consensus promoter**		
TAATACGACTCACTATTGGGAGA		
**Rho-independent terminators (hairpin stem in blue and loop in red)**		
AGTACACACATGGTCAACCTACGGGTTGGCCTTTTTGCGTTTT	5959	*g007*
AACTCACTATTGGGAGACCTTAACGGTTTCCCTTTGTTCGCTTC	9097	*g014*
GACTCACTATTGGGAGACACATTATGTTTCCCTTTAGTTTTAAC	19325	*g031*
AACTATATGAAACCCCTTGGGTGACCTTAACGGGTTACTTGAGGGGTTTTTTTTCTGAAA	21606	*g034*
TAACAGTAAATGCCTCTTTCAATACGACTCACTATTGAGAGGGGCtTTATTCATTAAGG	24653	*g036*

Table S5. Predicted promoter and terminator sequences and locations of Yersinia phage fPS-10

**Host RNAP promoters (-35 and -10 boxes)**	**5′-end pos.**	**Downstream gene (product)**
TCCACA $(N)_{13}$ ACCTATAGT	50	*g001* (hypothetical protein)
CTGAGT $(N)_{17}$ GAGTATCAT	361	*g001* (hypothetical protein)
TTAAAG $(N)_{14}$ TCTTAAAGT	684	*g001* (hypothetical protein)
ATGGAA $(N)_{12}$ GCTTATTAT	1002	*g002* (RNA polymerase),within *g001*
TTGCAG $(N)_{18}$ GGTTATATT	1314	*g002* (RNA polymerase)
**Phage RNAP promoters**		
TAATACAACTCACTATTGAGAGA	606	*g001* (hypothetical protein)
TAATAGAACTCACTATTGGGAGC	4098	*g003* (hypothetical protein)
TAATACAACTCACTATTGAGAAA	4372	*g004* (hypothetical protein)
TAATGGAACTCACTATTGGGAGA	6059	*g007* (phage protein)
TAATAGAACTCACTATTGAGAAC	7483	*g011*(ssDNA-binding protein)
TAATAGAACTCACTATTGGGAGA	9149	*g014* (homing endonuclease)
TAATACGACTCACTATTGAGAGG	15966	*g024* (phage protein)
TAATACGACTCACTATTGGGAGA	19376	*g031* (capsid protein)
TAATGCGACTCACTATTGGGAGA	20361	*g032* (capsid protein)
CAATACGACTCACTATTGAGAGG	24729	*g036* (core protein)
CAATATGACTCACTATTGGGAGT	32024	*g040* (tail fiber protein)
TAATACGACTCACTATTGAGAGA	38269	*g050* (phage protein)
**Consensus promoter**		
TAATACGACTCACTATTGGGAGA		
**Rho-independent terminators (hairpin stem in blue and loop in red)**		
AGTACACACATGGTCAACCTACGGGTTGGCCTTTTTGCGTTTT	6017	*g007*
GACTCACTATTGGGAGACACATTATGTTTCCCTTTAGTTTTAAC	19382	*g031*
AACTATATGAAACCCCTTGGGTGACCTTAACGGGTTACTTGAGGGGTTTTTTTTCTGAAA	21663	*g034*
TAACAGTAAATGCCTCTTTCAATACGACTCACTATTGAGAGGGGCtTTATTCATTAAGG	24710	*g036*

Table S6. Predicted promoter and terminator sequences and locations of Yersinia phage fPS-16

**Host RNAP promoters (-35 and -10 boxes)**	**5′-end pos.**	**Downstream gene (product)**
TCCACA $(N)_{14}$ ACCTATAGT	49	*g001* (hypothetical protein)
TTAAAG $(N)_{14}$ TCTTAAAGT	548	*g001* (hypothetical protein)
ATGGAA $(N)_{12}$ GCTTATTAT	866	*g002* (RNA polymerase),within *g001*
TTGCAG $(N)_{18}$ GGTTATATT	1178	*g002* (RNA polymerase)
**Phage RNAP promoters**		
TAATACAACTCACTATTGAGAGA	470	*g001* (hypothetical protein)
TAATAGAACTCACTATTGGGAGC	3962	*g003* (hypothetical protein)
TAATACAACTCACTATTGAGAAA	4236	*g004* (hypothetical protein)
TAATGGAACTCACTATTGGGAGA	5923	*g007* (phage protein)
TAATAGAACTCACTATTGAGAAC	7347	*g011* (ssDNA-binding protein)
TAATAGAACTCACTATTGGGAGA	9013	*g014* (homing endonuclease)
TAATACGACTCACTATTGAGAGG	15833	*g024* (phage protein)
TAATACGACTCACTATTGGGAGA	19244	*g031* (capsid protein)
TAATGCGACTCACTATTGGGAGA	20229	*g032* (capsid protein)
CAATACGACTCACTATTGAGAGG	24597	*g036* (core protein)
CAATATGACTCACTATTGGGAGT	31892	*g040* (tail fiber protein)
TAATACGACTCACTATTGAGAGA	38137	*g050* (phage protein)
**Consensus promoter**		
TAATACGACTCACTATTGGGAGA		
**Rho-independent terminators (hairpin stem in blue and loop in red)**		
AGTACACACATGGTCAACCTACGGGTTGGCCTTTTTGCGTTTT	5881	*g007*
GACTCACTATTGGGAGACACATTATGTTTCCCTTTAGTTTTAAC	19250	*g031*
AACTATATGAAACCCCTTGGGTGACCTTAACGGGTTACTTGAGGGGTTTTTTTTCTGAAA	21531	*g034*
TAACAGTAAATGCCTCTTTCAATACGACTCACTATTGAGAGGGGCtTTATTCATTAAGG	24578	*g036*

Table S7. Predicted promoter and terminator sequences and locations of Yersinia phage fPS-19

**Host RNAP promoters (-35 and -10 boxes)**	**5′-end pos.**	**Downstream gene (product)**
TCCACA (N)$_{14}$ ACCTATAGT	49	*g001* (hypothetical protein)
TTAAAG (N)$_{14}$ TCTTAAAGT	548	*g001* (hypothetical protein)
ATGGAA (N)$_{12}$ GCTTATTAT	866	*g002* (RNA polymerase),within *g001*
TTGCAG (N)$_{18}$ GGTTATATT	1178	*g002* (RNA polymerase)
**Phage RNAP promoters**		
TAATACAACTCACTATTGAGAGA	470	*g001* (hypothetical protein)
TAATAGAACTCACTATTGGGAGC	3962	*g003* (hypothetical protein)
TAATACAACTCACTATTGAGAAA	4236	*g004* (hypothetical protein)
TAATGGAACTCACTATTGGGAGA	5923	*g007* (phage protein)
TAATAGAACTCACTATTGAGAAC	7347	*g011* (ssDNA-binding protein)
TAATAGAACTCACTATTGGGAGA	9013	*g014* (homing endonuclease)
TAATACGACTCACTATTGAGAGG	15833	*g024* (phage protein)
TAATACGACTCACTATTGGGAGA	19244	*g031* (capsid protein)
TAATGCGACTCACTATTGGGAGA	20229	*g032* (capsid protein)
CAATACGACTCACTATTGAGAGG	24597	*g036* (core protein)
CAATACGACTCACTATTGGGAGT	31892	*g040* (tail fiber protein)
TAATACGACTCACTATTGAGAGA	38137	*g050* (phage protein)
**Consensus promoter**		
TAATACGACTCACTATTGGGAGA		
**Rho-independent terminators (hairpin stem in blue and loop in red)**		
AGTACACACATGGTCAACCTACGGGTTGGCCTTTTTGCGTTTT	5881	*g007*
GACTCACTATTGGGAGACACATTATGTTTCCCTTTAGTTTTAAC	19250	*g031*
AACTATATGAAACCCCTTGGGTGACCTTAACGGGTTACTTGAGGGGTTTTTTTTCTGAAA	21531	*g034*
TAACAGTAAATGCCTCTTTCAATACGACTCACTATTGAGAGGGGCtTTATTCATTAAGG	24578	*g036*

Table S8. Predicted promoter and terminator sequences and locations of Yersinia phage fPS-21

**Host RNAP promoters (-35 and -10 boxes)**	**5′-end pos.**	**Downstream gene (product)**
TCCACA $(N)_{15}$ ACCTATAGT	50	*g001* (hypothetical protein)
TTAAAG $(N)_{14}$ TCTTAAAGT	550	*g001* (hypothetical protein)
ATGGAA $(N)_{12}$ GCTTATTAT	868	*g002* (RNA polymerase),within *g001*
TTGCAG $(N)_{18}$ GGTTATATT	1180	*g002* (RNA polymerase)
**Phage RNAP promoters**		
TAATACAACTCACTATTGAGAGA	472	*g001* (hypothetical protein)
TAATAGAACTCACTATTGGGAGC	3964	*g003* (hypothetical protein)
TAATACAACTCACTATTGAGAAA	4238	*g004* (hypothetical protein)
TAATGGAACTCACTATTGGGAGA	5925	*g007* (phage protein)
TAATAGAACTCACTATTGAGAAC	7349	*g011* (ssDNA-binding protein)
TAATAGAACTCACTATTGGGAGA	9015	*g014* (homing endonuclease)
TAATACGACTCACTATTGAGAGG	15834	*g024* (phage protein)
TAATACGACTCACTATTGGGAGA	19245	*g031* (capsid protein)
TAATGCGACTCACTATTGGGAGA	20230	*g032* (capsid protein)
CAATACGACTCACTATTGAGAGG	24598	*g036* (core protein)
CAATACGACTCACTATTGGGAGT	31893	*g040* (tail fiber protein)
TAATACGACTCACTATTGAGAGA	38138	*g050* (phage protein)
**Consensus promoter**		
TAATACGACTCACTATTGGGAGA		
**Rho-independent terminators (hairpin stem in blue and loop in red)**		
AGTACACACATGGTCAACCTACGGGTTGGCCTTTTTGCGTTTT	5883	*g007*
GACTCACTATTGGGAGACACATTATGTTTCCCTTTAGTTTTAAC	19251	*g031*
AACTATATGAAACCCCTTGGGTGACCTTAACGGGTTACTTGAGGGGTTTTTTTTCTGAAA	21532	*g034*
TAACAGTAAATGCCTCTTTCAATACGACTCACTATTGAGAGGGGCtTTATTCATTAAGG	24579	*g036*

Table S9. Predicted promoter and terminator sequences and locations of Yersinia phage fPS-26

**Host RNAP promoters (-35 and -10 boxes)**	**5′-end pos.**	**Downstream gene (product)**
TTGTTA $(N)_{18}$ CCTTATCTT	86	*g001* (hypothetical protein)
TTAAAG $(N)_{14}$ TCTTAAAGT	654	*g001* (hypothetical protein)
ATGGAA $(N)_{12}$ GCTTATTAT	972	*g002* (RNA polymerase),within *g001*
TTGCAG $(N)_{18}$ GGTTATATT	1284	*g002* (RNA polymerase)
**Phage RNAP promoters**		
TAATACAACTCACTATTGAGAGA	576	*g001* (hypothetical protein)
TAATAGAACTCACTATTGGGAGC	4068	*g003* (hypothetical protein)
TAATACAACTCACTATTGAGAAA	4342	*g004* (hypothetical protein)
TAATGGAACTCACTATTGGGAGA	6029	*g007* (phage protein)
TAATAGAACTCACTATTGAGAAC	7453	*g011* (ssDNA-binding protein)
TAATAGAACTCACTATTGGGAGA	9119	*g014* (homing endonuclease)
TAATACGACTCACTATTGAGAGG	15934	*g024* (phage protein)
TAATACGACTCACTATTGGGAGA	19345	*g031* (capsid protein)
TAATGCGACTCACTATTGGGAGA	20330	*g032* (capsid protein)
CAATACGACTCACTATTGAGAGG	24698	*g036* (core protein)
CAATACGACTCACTATTGGGAGT	31993	*g040* (tail fiber protein)
TAATACGACTCACTATTGAGAGA	38238	*g050* (phage protein)
**Consensus promoter**		
TAATACGACTCACTATTGGGAGA		
**Rho-independent terminators (hairpin stem in blue and loop in red)**		
AGTAAACACATGGTCAACCTACGGGTTGGCCTTTTTGCGTTTT	5987	*g007*
GACTCACTATTGGGAGACACATTATGTTTCCCTTTAGTTTTAAC	19351	*g031*
AACTATATGAAACCCCTTGGGTGACCTTAACGGGTTACTTGAGGGGTTTTTTTTCTGAAA	21632	*g034*
TAACAGTAAATGCCTCTTTCAATACGACTCACTATTGAGAGGGGCtTTATTCATTAAGG	24679	*g036*

Table S10. Predicted promoter and terminator sequences and locations of Yersinia phage fPS-50

**Host RNAP promoters (-35 and -10 boxes)**	**5′-end pos.**	**Downstream gene (product)**
TTGTTA $(N)_{14}$ CCTTATCTT	109	*g001* (hypothetical protein)
ATGACA $(N)_{13}$ ATCTACAAG	413	*g001* (hypothetical protein)
TTAAAG $(N)_{9}$ AGTCATACT	721	*g001* (hypothetical protein)
ATGGAA $(N)_{12}$ GCTTATTAT	1107	*g002* (RNA polymerase),within *g001*
TTGCAG $(N)_{18}$ GGTTATATT	1419	*g002* (RNA polymerase)
**Phage RNAP promoters**		
TAATACAACTCACTATTGAGAGA	643	*g001* (hypothetical protein)
TAATAGAACTCACTATTGGGAGC	4203	*g003* (hypothetical protein)
TAATACAACTCACTATTGAGAAA	4819	*g005* (hypothetical protein)
TAATGGAACTCACTATTGGGAGA	6506	*g008* (phage protein)
TAATAGAACTCACTATTGAGAAC	7930	*g012* (ssDNA-binding protein)
TAATAGAACTCACTATTGGGAGA	9596	*g015* (homing endonuclease)
TAATACGACTCACTATTGAGAGG	16414	*g025* (phage protein)
TAATACGACTCACTATTGGGAGA	19818	*g030* (capsid protein)
TAATGCGACTCACTATTGGGAGA	20803	*g031* (capsid protein)
CAATACGACTCACTATTGAGAGG	25171	*g035* (core protein)
CAATACGACTCACTATTGGGAGT	32466	*g039* (tail fiber protein)
TAATACGACTCACTATTGAGAGA	38711	*g049* (phage protein)
**Consensus promoter**		
TAATACGACTCACTATTGGGAGA		
**Rho-independent terminators (hairpin stem in blue and loop in red)**		
AGTAAACACATGGTCAACCTACGGGTTGGCCTTTTTGCGTTTT	6464	*g008*
GACTCACTATTGGGAGACACTTTATGTTTCCCTTTAGTTTTAAC	19824	*g030*
AACTATATGAAACCCCTTGGGTGACCTTAACGGGTTACTTGAGGGGTTTTTTTTCTGAAA	22105	*g033*
TAACAGTAAATGCCTCTTTCAATACGACTCACTATTGAGAGGGGCtTTATTCATTAAGG	25152	*g035*

Table S11. Predicted promoter and terminator sequences and locations of Yersinia phage fPS-52

**Host RNAP promoters (-35 and -10 boxes)**	**5′-end pos.**	**Downstream gene (product)**
TTGTTA $(N)_{14}$ CCTTATCTT	109	*g001* (hypothetical protein)
TTAAAG $(N)_{9}$ AGTCATACT	677	*g001* (hypothetical protein)
ATGGAA $(N)_{12}$ GCTTATTAT	1086	*g002* (RNA polymerase),within *g001*
TTGCAG $(N)_{18}$ GGTTATATT	1398	*g002* (RNA polymerase)
**Phage RNAP promoters**		
TAATACAACTCACTATTGAGAGA	599	*g001* (hypothetical protein)
TAATAGAACTCACTATTGGGAGC	4182	*g003* (hypothetical protein)
TAATACAACTCACTATTGAGAAA	4798	*g005* (hypothetical protein)
TAATGGAACTCACTATTGGGAGA	6485	*g008* (phage protein)
TAATAGAACTCACTATTGAGAAC	7909	*g012* (ssDNA-binding protein)
TAATAGAACTCACTATTGGGAGA	9575	*g015* (homing endonuclease)
TAATACGACTCACTATTGAGAGG	16390	*g025* (phage protein)
TAATACGACTCACTATTGGGAGA	19794	*g030* (capsid protein)
TAATGCGACTCACTATTGGGAGA	20779	*g031* (capsid protein)
CAATACGACTCACTATTGAGAGG	25148	*g035* (core protein)
CAATACGACTCACTATTGGGAGT	32443	*g039* (tail fiber protein)
TAATACGACTCACTATTGAGAGA	38688	*g049* (phage protein)
**Consensus promoter**		
TAATACGACTCACTATTGGGAGA		
**Rho-independent terminators (hairpin stem in blue and loop in red)**		
AGTAAACACATGGTCAACCTACGGGTTGGCCTTTTTGCGTTTT	6443	*g008*
GACTCACTATTGGGAGACACTTTATGTTTCCCTTTAGTTTTAAC	19800	*g030*
AACTATATGAAACCCCTTGGGTGACCTTAACGGGTTACTTGAGGGGTTTTTTTTTCTGAA	22081	*g033*
TAACAGTAAATGCCTCTTTCAATACGACTCACTATTGAGAGGGGCtTTATTCATTAAGG	25129	*g035*

Table S12. Predicted promoter and terminator sequences and locations of Yersinia phage fPS-53

**Host RNAP promoters (-35 and -10 boxes)**	**5′-end pos.**	**Downstream gene (product)**
TTGTTA $(N)_{18}$ CCTTATCTT	77	*g001* (hypothetical protein)
TTAAAG $(N)_{14}$ TCTTAAAGT	614	*g001* (hypothetical protein)
GTGATT $(N)_{15}$ CAGTATAC	915	*g002* (kinase),within *g001*
TAGACA $(N)_{15}$ TGGTATACC	1260	*g003* (RNA polymerase),within *g002*
TTGAGA $(N)_{9}$ CCATATGAT	2154	*g003* (RNA polymerase)
**Phage RNAP promoters**		
TAATACAACTCACTATTGAGAGA	536	*g001* (hypothetical protein)
TAATACAACTCACTATTGAGAAA	5064	*g004* (hypothetical protein)
TAATGGAACTCACTATTGGGAGA	6751	*g007* (phage protein)
TAATAGAACTCACTATTGAGAAC	8175	*g011* (ssDNA-binding protein)
TAATAGAACTCACTATTGGGAGA	9841	*g014* (homing endonuclease)
TAATACGACTCACTATTGAGAGG	17082	*g025* (phage protein)
TAATACGACTCACTATTGGGAGA	20486	*g030* (capsid protein)
TAATGCGACTCACTATTGGGAGA	21471	*g031* (capsid protein)
CAATACGACTCACTATTGAGAGG	25838	*g035* (core protein)
CAATACGACTCACTATTGGGAGT	33133	*g039* (tail fiber protein)
TAATACGACTCACTATTGAGAGA	39609	*g049* (phage protein)
**Consensus promoter**		
TAATACGACTCACTATTGGGAGA		
**Rho-independent terminators (hairpin stem in blue and loop in red)**		
AGTACACACATGGTCAACCTACGGGTTGGCCTTTTTGCGTTTT	6720	*g007*
GACTCACTATTGGGAGACACTTTATGTTTCCCTTTAGTTTTAAC	20503	*g030*
AACTATATGAAACCCCTTGGGTGACCTTAACGGGTTACTTGAGGGGTTTTTTTCTGAAAG	22784	*g033*

Table S13. Predicted promoter and terminator sequences and locations of Yersinia phage fPS-54-ocr

**Host RNAP promoters (-35 and -10 boxes)**	**5′-end pos.**	**Downstream gene (product)**
TCCACA $(N)_{14}$ ACCTATAGT	49	*g001* (hypothetical protein)
TTAAAG $(N)_{15}$ TCTTAAAGT	618	*g001* (hypothetical protein)
GTGATT $(N)_{15}$ CAGTATACT	919	*g002* (kinase),within *g001*
TAGACA $(N)_{15}$ TGGTATACC	1264	*g003* (RNA polymerase),within *g002*
TTTACA $(N)_{18}$ GATTATTAT	1623	*g003* (RNA polymerase),within *g002*
TTGAGA $(N)_{14}$ CCATATGAT	2158	*g003* (RNA polymerase)
**Phage RNAP promoters**		
TAATACAACTCACTATTGAGAGA	540	*g001* (hypothetical protein)
TAATACAACTCACTATTGAGAAA	5068	*g004* (hypothetical protein)
TAATGGAACTCACTATTGGGAGA	6755	*g007* (phage protein)
TAATAGAACTCACTATTGAGAAC	8179	*g011* (ssDNA-binding protein)
TAATAGAACTCACTATTGGGAGA	9845	*g014* (homing endonuclease)
TAATACGACTCACTATTGAGAGG	17086	*g025* (phage protein)
TAATACGACTCACTATTGGGAGA	20091	*g030* (capsid protein)
TAATGCGACTCACTATTGGGAGA	21076	*g031* (capsid protein)
CAATACGACTCACTATTGAGAGG	25454	*g035* (core protein)
CAATACGACTCACTATTGGGAGT	32749	*g039* (tail fiber protein)
TAATACGACTCACTATTGAGAGA	39233	*g049* (phage protein)
**Consensus promoter**		
TAATACGACTCACTATTGAGAGA		
**Rho-independent terminators (hairpin stem in blue and loop in red)**		
AGTACACACATGGTCAACCTACGGGTTGGCCTTTTTGCGTTTT	6713	*g007*
ACTCACTATTGGGAGACACTTTATGTTTCCTTTTAGTTTTAA	20197	*g030*
AACTATATGAAACCCCTTGGGTGACCTTAACGGGTTACTTGAGGGGTTTTTTTTTCTGAA	22477	*g033*
ATTATGGAGTACCGTGACATCATGGTTTTACGCTATG	25845	*g036*

Table S14. Predicted promoter and terminator sequences and locations of Yersinia phage fPS-59

**Host RNAP promoters (-35 and -10 boxes)**	**5′-end pos.**	**Downstream gene (product)**
CTCTCA $(N)_{14}$ ACCTATAGT	39	*g001* (hypothetical protein)
TTTATA $(N)_{16}$ AGTCATACT	624	*g002* (hypothetical protein)
GTGATT $(N)_{15}$ CAGTATACT	944	*g003* (RNA polymerase),within *g002*
TGGGAA $(N)_{18}$ AGTTATATT	1616	*g003* (RNA polymerase)
**Phage RNAP promoters**		
TAATACAACTCACTATTGAGAGA	576	*g002* (hypothetical protein)
TAATACAACTCACTATTGAGAAA	4398	*g004* (hypothetical protein)
TAATGGAACTCACTATTGGGAGA	6085	*g007* (phage protein)
TATTAGAACTCACTATTGAGAAC	7509	*g011* (ssDNA-binding protein)
TATTAGAACTCACTTTGGGAGA	9666	*g015* (homing endonuclease)
TAATACGACTCACTATTGAGAGG	16897	*g027* (phage protein)
TAGTACGACTCACTATTGGGAGA	19583	*g030* (capsid protein)
TAATGCGACTCACTATTGGGAGA	20568	*g031* (capsid protein)
CAATACGACTCACTATTGAGAGG	24935	*g035* (core protein)
CAACACGACTCACTATTGGGAGT	32230	*g039* (tail fiber protein)
TAATACGACTCACTATTGAGAGA	37557	*g046* (Phage protein)
**Consensus promoter**		
TAATACGACTCACTATTGGGAGA		
**Rho-independent terminators (hairpin stem in blue and loop in red)**		
AGTACACACATGGTCAACCTACGGGTTGACCTTTTTGCGTTTT	6043	*g007*
GACTCACTATTGGGAGACACTTTATGTTTCCCTTTAGTTTTAAC	19589	*g030*
AACTATATGAAACCCCTTGGGTGACCTTAACGGGTTACTTGAGGGGTTTTTTTCTGAAAG	21870	*g033*
TAACAGTAAATGCCTCTTTCAATACGACTCACTATTGAGAGGGGCtTTATTCATTAAGG	24916	*g035*

Table S15. Predicted promoter and terminator sequences and locations of Yersinia phage fPS-64

**Host RNAP promoters (-35 and -10 boxes)**	**5′-end pos.**	**Downstream gene (product)**
TTGTTA $(N)_{18}$ CCTTATCTT	85	*g001* (hypothetical protein)
CTGAGT $(N)_{18}$ GAGTATCAT	494	*g001* (hypothetical protein)
TTAAAG $(N)_{14}$ TCTTAAAGT	808	*g001* (hypothetical protein)
ATGGAA $(N)_{12}$ GCTTATTAT	1126	*g002* (RNA polymerase),within *g001*
TTGCAG $(N)_{18}$ GGTTATATT	1438	*g002* (RNA polymerase)
**Phage RNAP promoters**		
TAATACAACTCACTATTGAGAGA	730	*g001* (hypothetical protein)
TAATAGAACTCACTATTGGGAGC	4222	*g003* (hypothetical protein)
TAATACAACTCACTATTGAGAAA	4496	*g004* (hypothetical protein)
TAATGGAACTCACTATTGGGAGA	6183	*g007* (Phage protein)
TAATAGAACTCACTATTGAGAAC	7607	*g011* (ssDNA-binding protein)
TAATAGAACTCACTATTGGGAGA	9273	*g014* (homing endonuclease)
TAATACGACTCACTATTGAGAGG	16089	*g024* (phage protein)
TAATACGACTCACTATTGGGAGA	19500	*g031* (capsid protein)
TAATGCGACTCACTATTGGGAGA	20485	*g032* (capsid protein)
CAATACGACTCACTATTGAGAGG	24854	*g036* (core protein)
CAATACGACTCACTATTGGGAGT	32149	*g040* (phage tail fiber)
TAATACGACTCACTATTGAGAGA	38394	*g050* (phage protein)
**Consensus promoter**		
TAATACGACTCACTATTGGGAGA		
**Rho-independent terminators (hairpin stem in blue and loop in red)**		
AGTAAACACATGGTCAACCTACGGGTTGGCCTTTTTGCGTTTT	6141	*g007*
GACTCACTATTGGGAGACACATTATGTTTCCCTTTAGTTTTAAC	19805	*g031*
AACTATATGAAACCCCTTGGGTGACCTTAACGGGTTACTTGAGGGGTTTTTTTTTCTGAA	22086	*g034*
TAACAGTAAATGCCTCTTTCAATACGACTCACTATTGAGAGGGGCtTTATTCATTAAGG	24835	*g036*

Table S16. Predicted promoter and terminator sequences and locations of Yersinia phage fPS-85

**Host RNAP promoters (-35 and -10 boxes)**	**5′-end pos.**	**Downstream gene (product)**
TTGTTA $(N)_{18}$ CCTTATCTT	77	*g001* (hypothetical protein)
TTAAAG $(N)_{14}$ TCTTAAAGT	614	*g001* (hypothetical protein)
GTGATT $(N)_{15}$ CAGTATACT	915	*g002* (kinase),within *g001*
TAGACA $(N)_{15}$ TGGTATACC	1260	*g003* (RNA polymerase),within *g002*
TTGAGA $(N)_{14}$ CCATATGAT	2154	*g003* (RNA polymerase)
**Phage RNAP promoters**		
TAATACAACTCACTATTGAGAGA	536	*g001* (hypothetical protein)
TAATACAACTCACTATTGAGAAA	5064	*g004* (hypothetical protein)
TAATGGAACTCACTATTGGGAGA	6751	*g007* (phage protein)
TAATAGAACTCACTATTGAGAAC	8175	*g011* (ssDNA-binding protein)
TAATAGAACTCACTATTGGGAGA	9841	*g014* (primase/helicase)
TAATACGACTCACTATTGAGAGG	17082	*g024* (phage protein)
TAATACGACTCACTATTGGGAGA	20486	*g029* (capsid protein)
TAATGCGACTCACTATTGGGAGA	21471	*g030* (capsid protein)
CAATACGACTCACTATTGAGAGG	25838	*g034* (core protein)
CAATACGACTCACTATTGGGAGT	33133	*g038* (phage tail fiber)
TAATACGACTCACTATTGAGAGA	39609	*g048* (phage protein)
**Consensus promoter**		
TAATACGACTCACTATTGGGAGA		
**Rho-independent terminators (hairpin stem in blue and loop in red)**		
AGTACACACATGGTCAACCTACGGGTTGGCCTTTTTGCGTTTT	6410	*g007*
GACTCACTATTGGGAGACACTTTATGTTTCCCTTTAGTTTTAAC	20193	*g029*
AACTATATGAAACCCCTTGGGTGACCTTAACGGGTTACTTGAGGGGTTTTTTTCTGAAAG	22474	*g032*

Table S17. Predicted promoter and terminator sequences and locations of Yersinia phage fPS-86

**Host RNAP promoters (-35 and -10 boxes)**	**5′-end pos.**	**Downstream gene (product)**
TTGTTA $(N)_{18}$ CCTTATCTT	96	*g001* (hypothetical protein)
TTAAAG $(N)_{14}$ TCTTAAAGT	584	*g001* (hypothetical protein)
ATGGAA $(N)_{12}$ GCTTATTAT	902	*g002* (RNA polymerase),within *g001*
TTGCAG $(N)_{18}$ GGTTATATT	1214	*g002* (RNA polymerase)
**Phage RNAP promoters**		
TAATACAACTCACTATTGAGAGA	506	*g001* (hypothetical protein)
TAATAGAACTCACTATTGGGAGC	3998	*g003* (hypothetical protein)
TAATACAACTCACTATTGAGAAA	4272	*g004* (hypothetical protein)
TAATGGAACTCACTATTGGGAGA	5959	*g007* (Phage protein)
TAATAGAACTCACTATTGAGAAC	7383	*g011* (ssDNA-binding protein)
TAATAGAACTCACTATTGGGAGA	9049	*g014* (homing endonuclease)
TAATACGACTCACTATTGAGAGG	15863	*g024* (phage protein)
TAATACGACTCACTATTGGGAGA	19274	*g031* (capsid protein)
TAATGCGACTCACTATTGGGAGA	20259	*g032* (capsid protein)
CAATACGACTCACTATTGAGAGG	24626	*g036* (core protein)
CAATACGACTCACTATTGGGAGT	31921	*g040* (tail fiber protein)
TAATACGACTCACTATTGAGAGA	38166	*g050* (phage protein)
**Consensus promoter**		
TAATACGACTCACTATTGGGAGA		
**Rho-independent terminators (hairpin stem in blue and loop in red)**		
AGTAAACACATGGTCAACCTACGGGTTGGCCTTTTTGCGTTTT	5917	*g007*
GACTCACTATTGGGAGACACATTATGTTTCCCTTTAGTTTTAAC	19280	*g031*
AACTATATGAAACCCCTTGGGTGACCTTAACGGGTTACTTGAGGGGTTTTTTTCTGAAAG	21561	*g034*
TAACAGTAAATGCCTCTTTCAATACGACTCACTATTGAGAGGGGCtTTATTCATTAAGG	24607	*g036*

Table S18. Predicted promoter and terminator sequences and locations of Yersinia phage fPS-89

**Host RNAP promoters (-35 and -10 boxes)**	**5′-end pos.**	**Downstream gene (product)**
TTGTTA $(N)_{18}$ CCTTATCTT	76	*g001* (hypothetical protein)
TTAAAG $(N)_{14}$ TCTTAAAGT	569	*g001* (hypothetical protein)
GTGATT $(N)_{15}$ CAGTATACT	870	*g002* (kinase),within *g001*
TAGACA $(N)_{15}$ TGGTATACC	1215	*g003* (RNA polymerase),within *g002*
TTGAGA $(N)_{15}$ CCATATGAT	2109	*g003* (RNA polymerase)
**Phage RNAP promoters**		
TAATACAACTCACTATTGAGAGA	491	*g001* (hypothetical protein)
TAATACAACTCACTATTGAGAAA	5019	*g004* (hypothetical protein)
TAATGGAACTCACTATTGGGAGA	6706	*g007* (phage protein)
TAATAGAACTCACTATTGAGAAC	8130	*g011* (ssDNA-binding protein)
TAATAGAACTCACTATTGGGAGA	9796	*g014* (homing endonuclease)
TAATACGACTCACTATTGAGAGG	17037	*g025* (phage protein)
TAATACGACTCACTATTGGGAGA	20441	*g030* (capsid protein)
TAATGCGACTCACTATTGGGAGA	21426	*g031* (capsid protein)
CAATACGACTCACTATTGAGAGG	25793	*g035* (core protein)
CAATACGACTCACTATTGGGAGT	33088	*g039* (tail fiber protein)
TAATACGACTCACTATTGAGAGA	39564	*g049* (phage protein)
**Consensus promoter**		
TAATACGACTCACTATTGGGAGA		
**Rho-independent terminators (hairpin stem in blue and loop in red)**		
AGTACACACATGGTCAACCTACGGGTTGGCCTTTTTGCGTTTT	6664	*g007*
GACTCACTATTGGGAGACACTTTATGTTTCCCTTTAGTTTTAAC	20447	*g030*
AACTATATGAAACCCCTTGGGTGACCTTAACGGGTTACTTGAGGGGTTTTTTTCTGAAAG	22728	*g033*

Table S19. Repeats in the fPS-phage genomes between left TR and phage promoter P1. See Figure S3.

Phage	Rep1[a]	Rep2[b]	Rep3[c]	Rep4[d]	Sum
fPS-50	9	0	6	0	15
fPS-52	11	1	5	0	17
fPS-21	3	2	6	0	11
fPS-16	3	2	6	0	11
fPS-9	6	0	10	0	16
fPS-64	26	4	4	0	34
fPS-10	9	0	16	0	25
fPS-26	16	0	4	0	20
fPS-19	3	2	6	0	11
fPS-7	3	3	5	0	11
fPS-86	5	1	7	0	13
fPS-53	5	0	13	0	18
fPS-85	5	0	13	0	18
fPS-89	2	0	11	0	13
fPS-54-ocr	5	0	13	0	18
fPS-59	11	0	0	7	18

[a] Rep1 ACCACTGAGTA
[b] Rep2 ACCATTGAGTA
[c] Rep3 TCATTGAGTA
[d] Rep4 TCATTAAGGA

Table S20. Repeats in the fPS-phage genomes between right TR and phage promoter P12. See Figure S3.

Phage	Rep5[a]	Rep6[b]	Rep7[c]	Rep8[d]	Rep9[e]	Rep10[f]	Sum
fPS-50	5	1	0	2	0	1	9
fPS-52	9	6	0	2	0	1	18
fPS-21	0	1	0	2	0	1	4
fPS-16	4	0	0	1	0	3	8
fPS-9	3	0	0	0	0	2	5
fPS-64	0	3	0	8	0	5	16
fPS-10	4	0	0	0	0	2	6
fPS-26	0	1	0	4	0	3	8
fPS-19	2	1	0	1	0	1	5
fPS-7	4	0	0	0	0	2	6
fPS-86	0	2	0	4	0	2	8
fPS-53	0	0	4	4	0	0	8
fPS-85	0	0	3	3	0	0	6
fPS-89	0	0	4	4	0	0	8
fPS-54-ocr	0	0	4	4	0	0	8
fPS-59	0	3	0	0	3	0	6

[a] Rep5 CTCTAAGGAT
[b] Rep6 ACCTAATGGTA
[c] Rep7 GATGTACTCTAAGATACCTAAT
[d] Rep8 ACTCTAAGATACCTAAT
[e] Rep9 TACCTAATGGTACTTGAAGA
[f] Rep10 ACCTAATGATGCA
